# The introduction of dengue follows transportation infrastructure changes in the state of Acre, Brazil: A network-based analysis

**DOI:** 10.1371/journal.pntd.0006070

**Published:** 2017-11-17

**Authors:** Raquel Martins Lana, Marcelo Ferreira da Costa Gomes, Tiago França Melo de Lima, Nildimar Alves Honório, Cláudia Torres Codeço

**Affiliations:** 1 Fiocruz, Pós-Graduação em Epidemiologia em Saúde Pública, Escola Nacional de Saúde Pública Sérgio Arouca (ENSP), Rio de Janeiro, RJ, Brazil; 2 Fiocruz, Programa de Computação Científica (PROCC), Rio de Janeiro, RJ, Brazil; 3 Laboratório de Engenharia e Desenvolvimento de Sistemas (LEDS), Departamento de Computação e Sistemas (DECSI), Instituto de Ciências Exatas e Aplicadas (ICEA), Universidade Federal de Ouro Preto (UFOP), João Monlevade, MG, Brazil; 4 Fiocruz, Instituto Oswaldo Cruz (IOC), Laboratório de Mosquitos Transmissores de Hematozoários, Rio de Janeiro, RJ, Brazil; 5 Fiocruz, Núcleo Operacional Sentinela de Mosquitos Vetores (Nosmove), Rio de Janeiro, RJ, Brazil; Centers for Disease Control and Prevention, Puerto Rico, UNITED STATES

## Abstract

Human mobility, presence and passive transportation of *Aedes aegypti* mosquito, and environmental characteristics are a group of factors which contribute to the success of dengue spread and establishment. To understand this process, we assess data from dengue national and municipal basins regarding population and demographics, transportation network, human mobility, and *Ae. aegypti* monitoring for the Brazilian state of Acre since the first recorded dengue case in the year 2000 to the year 2015. During this period, several changes in Acre’s transport infrastructure and urbanization have been started. To reconstruct the process of dengue introduction in Acre, we propose an analytic framework based on concepts used in malaria literature, namely vulnerability and receptivity, to inform risk assessments in dengue-free regions as well as network theory concepts for disease invasion and propagation. We calculate the probability of dengue importation to Acre from other Brazilian states, the evolution of dengue spread between Acrean municipalities and dengue establishment in the state. Our findings suggest that the landscape changes associated with human mobility have created favorable conditions for the establishment of dengue virus transmission in Acre. The revitalization of its major roads, as well as the increased accessibility by air to and within the state, have increased dengue vulnerability. Unplanned urbanization and population growth, as observed in Acre during the period of study, contribute to ideal conditions for *Ae. aegypti* mosquito establishment, increase the difficulty in mosquito control and consequently its local receptivity.

## Author summary

The present ecological study is aimed at characterizing introduction and spread of dengue in the state of Acre, located in Brazil’s north region. We use sociodemographic and entomological data from the year 2000, when the first case was registered in Acre, up to 2015, when it was already endemic in most of the state. We use a methodological framework based on well-established concepts in malaria literature, namely vulnerability and receptivity, to assess dengue introduction and propagation in the study area. We make use of effective reproduction number as an empirical indicator of dengue outbreak spatiotemporal patterns, as well as complex network theory concepts applied to human mobility and transport infrastructure changes to address dengue importation probability from other Brazilian states and its circulation within Acrean municipalities. On the one hand, we show that the increase of airline passengers, coupled with observed dengue incidence in Brazil, has steadily increased Acre’s vulnerability to dengue introduction, while transport infrastructure development has facilitated its spread within the state. On the other hand, socio-demographic changes aimed at urbanization and economic development have increased receptivity to its main vector, the *Aedes aegypti* mosquito. The combination of those two factors ultimately leads to the establishment of the virus in Acre.

## Introduction

The worldwide spread of dengue fever has been attributed to human mobility [1] as well as the passive transportation of its vector *Aedes aegypti*, a mosquito with autonomous flying capacity [2, 3]. According to the literature, the global spread of dengue from Africa to Asia and the Americas was historically caused by the periodic human voyages across the oceans [4, 5]. Godding [6] further described outbreaks of dengue aboard ships. More recently, in a local survey in Iquitos, Peru, Stoddard et al. [1] showed that the movement of humans to places where contact with mosquitoes was probable was determinant for the transmission of dengue, regardless of the distance between people’s place of residence. On a collective scale, spatial patterns of dengue distribution are determined by the social networks, since an individual’s routine movements are similar to those of their contacts [1].

More recently, increased air transportation also contributed to dengue spread, carrying both infected humans and mosquitoes to distant places. There are reports of catching *Ae. aegypti* within aircraft that landed in cities where there were no records of dengue cases [7]. Several studies of disease dispersion have used airline database and human mobility networks coupled with epidemiological models to provide mathematical frameworks for the study of spatiotemporal dispersion [8, 9], risk assessment of case importation during outbreaks [10] and estimation of potential invasion routes [11, 12]. Such frameworks have been applied to a variety of illnesses such as the 1968-69 Hong Kong [8] and the 2009 H1N1 influenza pandemics [13], the 2013 MERS-CoV epidemic [14], the 2014 Ebola outbreak in West Africa [15–17], and the 2016 Zika outbreak in the Americas [18], for instance.

In Brazil, reports of diseases compatible with dengue date back to the 19^th^ century [19, 20], but its circulation was interrupted in conjunction with the urban yellow fever after the intense *Ae. aegypti* eradication campaigns in the early 20^th^ century. After the re-invasion of the mosquito in the 1970s, the reintroduction of dengue viruses was laboratory confirmed in 1982 in the state of Roraima. In 1986, after four years without records of dengue cases, a DENV-1 epidemic settled in Rio de Janeiro with fatal cases confirmed in the laboratory [21, 22]. Since then, dengue became endemic in Brazil, with the circulation of all four serotypes, and mostly concentrated in the southeast region.

Among the Brazilian states, the northwesternmost state of Acre, in the Amazon region, was the last to register dengue cases. The Amazon region is one of the last frontiers of development in Brazil. At its western extreme, Acre occupies a strategic, but underdeveloped, place of integration in South America due to its geographical position in relation to the Pacific Ocean and its triple border with Peru and Bolivia. In the last 15 years, Acre has been the target of diverse and intensive government programs aimed at boosting its development. Examples of such programs are agrarian reform with the creation of several settlements; paving of roadways and expansion of airports [23, 24]; the stimulus to new economic activities; and strengthening the exchange of commodities between Brazil and the Pacific countries by means of the Pacific road, completed in 2010. In response to these investments, the population of some municipalities doubled from the year 2000 to 2010, and the percentage of urbanization increased in 20 of its 22 municipalities [25]. It was during this period that the introduction and spread of the dengue virus in the state was observed, almost 20 years after its introduction and establishment in the country [21].

We hypothesize that the rate and pattern of dengue introduction and establishment in the 22 municipalities of Acre were directly affected by the fast changes in infrastructure and urbanization. In this paper, we reconstruct the history of dengue introduction and establishment in Acre, linking the epidemiological events to the evolution of human mobility within the state, and between the state and the rest of the country. In this process, we propose an analytical framework to inform (to be used in) risk assessments in other dengue-free regions under similar development pressure.

The goal of this study was to assess how the risk of dengue establishment varied from 2000 to 2015 in the 22 cities of Acre, as the region changed in response to significant investments in infrastructure and migration. For this purpose, we: 1) assessed how vulnerability (defined in the next section) to dengue importation from outside the state changed during the study period and identified the most likely sources of dengue to Acre; 2) assessed the relationship between vulnerability to dengue within Acre, measured by rate of establishment, and municipalities’ centrality in terms of human mobility; 3) assessed available evidence on Acre’s receptivity to dengue (defined in the next section) and how it changed during the study period.

### Conceptual framework

The successful invasion of a parasite into a new place is the result of the interaction between two players: the invading species and the invaded community. In the context of dengue, the invaded community is composed of two main species: human hosts and mosquitoes of the species *Ae. aegypti* whose interaction is further affected by environmental factors (climate, urbanization, human behavior). The invading species under consideration, dengue viruses, cannot establish itself in new areas unless the receiving community presents certain conditions to sustain autochthonous virus transmission. More formally, the emergence of dengue fever in a new region depends on the occurrence of two major events: (i) the arrival of at least one infected individual in the city and (ii) the subsequent positive growth of autochthonous dengue cases (i.e., disease establishment) [26].

The sustained growth of autochthonous cases depends on “*the abundant presence of vectors and the existence of other ecological and climatic factors favoring transmission*”, which is the concept of receptivity found in the malaria literature [27]. If an area is receptive, then the arrival of the virus by imported cases or vectors has a probability of triggering sustained local transmission. Transmission is then measured by the effective reproductive number, *R*_*t*_, defined as the expected number of secondary human cases that would result from the arrival of a single infected individual in a susceptible population. A reproductive number greater than one suggests epidemic growth.

The larger the influx of imported cases the higher the probability of establishment given the necessary environmental conditions. In the malaria literature, this exposure to imported cases is referred to as “Vulnerability” defined as “*either proximity to endemic areas or…the frequent influx of infected individuals or groups and/or infective anopheles*” [28]. The same concept can be applied to dengue epidemiology. Dengue influx might be measured directly from the number of cases that can be traced to outside areas (imported cases). In situations where the origin of the cases is difficult to ascertain, one can estimate the number of imported cases from statistics of transportation and prevalence in passengers’ origins [26].

## Materials and methods

### Study area

**Fig 1:**
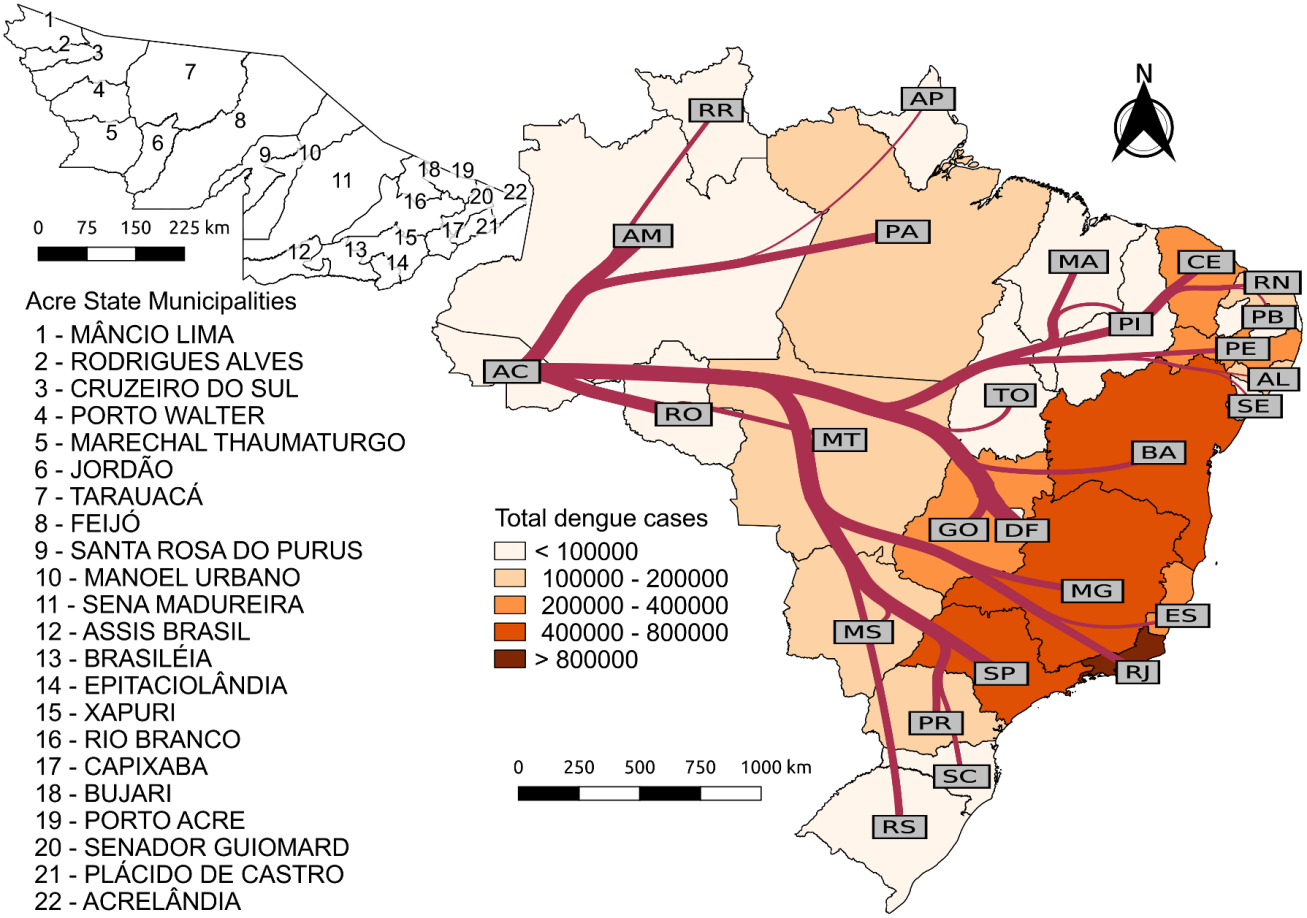
Dengue cases in Brazilian states and airline flow to the state of Acre. The map is colored according to the total number of reported dengue cases in each state during the period of this study (from dengue epidemiological year 2001/2002 to 2011/2012). The left panel shows the municipalities of the state of Acre. The network over the country map on the right panel represents the flow of airline passengers to Acre from each state. Edges widths are proportional to the logarithm of the total number of passengers from 2001/2002 to 2011/2012. The underlying shapefiles with political boundaries of Brazilian states and municipalities used to build this figure are publicly available and free to use at *Instituto Brasileiro de Geografia e Estatística* (IBGE, Brazilian Institute of Geography and Statistics) website http://downloads.ibge.gov.br/downloads_geociencias.htm.

The state of Acre (AC) is located in the north region of Brazil, bordering the countries of Peru and Bolivia, and the Amazonas and Rondônia states in Brazil (see Fig 1 for geographical reference). The total area is 164,122 km^2^ with a population of 733,559 inhabitants and a population density of 4.47 hab/km^2^ [25]. The state has 22 municipalities, the most populous being the capital Rio Branco and Cruzeiro do Sul, with 336,038 and 78,507 inhabitants according to 2010 Census [25], respectively. The Acrean climate is warm and humid equatorial, which is characterized by high temperatures with small annual fluctuations, high pluviometric precipitation indices and high relative humidity. The temperature is approximately uniform throughout the state, with an annual average around 24,5°C, and maximum around 32°C [24].

### Data

#### Dengue national data

Monthly counts of reported cases of dengue per Brazilian state, from 2001 to 2012, were obtained from *Departamento de Informática do Sistema Único de Saúde* (DataSUS, Department of Informatics of Universal Health System, http://datasus.saude.gov.br/). The accumulated number of cases in each state during the period of study is shown in Fig 1.

#### Dengue municipal data

Weekly counts of reported cases of dengue per Acrean municipality, from 2000 to 2015, were provided by the Brazilian Ministry of Health. Dengue reporting is mandatory in Brazil, but most cases are defined using clinical-epidemiological criteria only.

#### Definition of dengue epidemiological year

Since dengue reaches higher activity levels during the summer (Dec to March), it is more convenient to define the epidemiological year as the period between July of a year and June of the next year, as opposed to the standard calendar year.

#### *Aedes aegypti* data

Available data regarding the presence of *Ae. aegypti* in the 22 municipalities of Acre was obtained from the Ministry of Health (MH) and internet bulletins. Although MH has implemented the household larval survey (LI) and the Rapid Assessment of Infestation by *Ae. aegypti* (LIR*Aa*) since 1996, not all municipalities adhered to the protocol at the same time. We searched the available information to identify the year in which the presence of the mosquito was confirmed for the first time in each municipality. It is important to note, however, that this information can be incomplete for some cities, and the first detection does not necessarily imply that the mosquito was not present before.

#### Population and demographic data

The population size per Brazilian state from 2001 to 2012 was obtained from *Instituto Brasileiro de Geografia e Estatística* (IBGE, Brazilian Institute of Geography and Statistics) [25, 29]. The population count in rural and urban areas per municipality in Acre, in 2000 and 2010, as well as information on the proportion of households with access to public services and other demographic variables were obtained from [25].

#### Transportation network

To describe the Acrean transportation system, data regarding the availability and status of roads, waterways, and airways was obtained from the Ministry of Transport (http://www.transportes.gov.br), *Agência Nacional de Aviação Civil* (ANAC, National Civil Aviation Agency, http://www.anac.gov.br) and *Associação dos Municípios do Acreanos* (AMAC, Association of Acrean Municipalities, http://www.amac-acre.com.br). Description of road status was obtained by request to the *Departamento de Estradas e Rodagens do Acre* (DERACRE, Department of Roads and Roadways of Acre) and by consulting the website of the *Programa de Aceleração do Crescimento* (PAC, Growth Acceleration Program, http://www.pac.gov.br/). The connections between municipalities—i.e., the network edges—were classified according to the presence and status of roads, waterways, and airways. In the cases where municipalities were connected by more than one modal, this was taken into account by including all corresponding edges. Road status was tentatively classified year by year. However, since not all information was recorded every year, it is only an approximation. The combination of modal type and road condition resulted in the following categories: (1) unpaved road, (2) paved road and (3) road under maintenance; (4) waterway; and (5) airway. The third category encompassed roads under restoration, under construction and mixed conditions.

#### Flight data

To describe the flow of individuals to Acre, airline data from 2001 to 2012 was provided by ANAC. This dataset contains information on the origin-destination for passengers on direct flights (with or without stoppage). For those on connecting flights, ANAC provides the number of travelers *in transit* per airport pair. We developed a methodology to combine the data regarding direct flights and passengers in transit to estimate the total number of passengers that travel between all possible airport pairs (see S1 Appendix). The total number of passengers to Acre by state of origin during the period of study is depicted in Fig 1.

#### Commutation data (Mobility)

To obtain an estimate of the flow of individuals between municipalities in Acre, we used data from the 2010 Brazilian Census [30]. In this dataset, respondents were asked to provide the country, state, and municipality of work/study. A fraction of them did not provide full information, that is, they mentioned working outside their municipality of residence, but with no further specification of state or municipality; some only provided destination state, but no specification of the municipality. To compensate for that, for each city of residence we aggregated all entries with the same level of information, (i) displacement only, (ii) destination state only, and (iii) complete information. With that, we proportionally assigned a destination municipality based on the distribution for the particular subset of the entries with full information.

The *mobility network* is built from the combination of commutation and flight data. In this network, each municipality is represented by a node while edges represent the average daily flow of individuals between pairs. To estimate this quantity, we summed the estimated daily number of residents moving from one municipality to another for work and/or study over the year, given by the commutation data, with the average daily number of flight passengers in 2010.

### Assessing how Acre’s vulnerability to dengue importation changed from 2001 to 2012

Within our conceptual framework, vulnerability was defined in terms of the exposure of the focal population to imported dengue cases. Here, we measured vulnerability as the expected probability of dengue importation from other Brazilian states into Acre, by air transportation.

The usage of national airline passengers database is of great interest for the study of human interaction dynamics within a country, particularly for the study of potential disease transmission routes. The flow of individuals between different regions allows agents that became infected in one area to carry the pathogen to another area within his/her path [8, 11, 17, 18, 26, 31]. This mechanism serves as a driver for the (re-)introduction of pathogens throughout the national territory. In this context, we analyzed Brazilian airline grid to estimate passenger flow between Brazilian states and how this potential risk for the spreading of dengue to Acre varied since the year 2000.

From the Brazilian airline database collected from 2001 to 2012, we estimated the daily probability of an individual from state *i* traveling to state *j* for each month *m*, *π*_*ij*,*m*_ (see S1 Appendix). Coupling this probability with the reported number of dengue cases in each state in the corresponding time window, we estimated the risk of each one state being the source of an imported dengue case to any other in the Brazilian territory. This framework allows us to estimate the most probable routes of case importation in each year to Acre.

Say we have *k*_*i*,*m*_ dengue cases reported in state *i* in month *m*. Each *k* of the *k*_*i*,*m*_ infected individuals stays *τ*_*k*_ days as infectious, with an average of 4.5 [1.9–7.9] days [32]. Given the daily probability of travel *π*_*ij*,*m*_, *τ*_*k*_ is also the number of monthly trials for the probability of an infected individual boarding a flight during his infectious period. Combining this information, the monthly case importation probability *p*_*ij*,*m*_ can be estimated as

$$\begin{array}{cc} p_{ij,m} & =1-{\left(1-\pi_{ij,m}\right)}^{\sum_{k=1}^{k_{i,m}}\tau_{k}}. \end{array}$$ (1)

Following the same rationale, the yearly probability of dengue importation, *P*_*ij*,*s*_, at dengue epidemiological year *s*, can be estimated by aggregating over all months of the corresponding epidemiological year, that is

$$\begin{array}{cc} P_{ij,s} & =1-\prod_{m}(1-p_{ij,m}). \end{array}$$ (2)

This construction allows us to provide a ranking of states by their probability of exporting a dengue-infected individual to Acre, that is, the most probable sources of case importation at any given epidemiological year. It also allows us to estimate the source-independent yearly dengue importation probability, that is, the probability of at least one importation to Acre in a given year regardless of its state of origin, which can be written as:

$$\begin{array}{cc} P_{j,s} & =1-\prod_{i}(1-P_{ij,s}) \end{array}$$ (3)

This quantity describes the temporal dynamics of the Acre’s overall exposure to dengue-infected individuals. To take into account the uncertainty regarding the length of the infectious period, the results shown correspond to the average over 1000 simulations of this process using a gamma distribution with mean of 4.5 and 95% confidence interval of [1.9–7.9] for the infectious period of each reported case.

### Establishment of dengue transmission in the Acrean cities

The earliest evidence of the onset of disease establishment is the occurrence of autochthonous cases, which are ascertained based on investigations of the travel history of the patients. Secondly, the presence of clusters or transmission chains is indicative of further transmission. Thirdly, if the transmission is sustained, the incidence curve will increase at an exponential (or subexponential) rate, characterizing an epidemic [33]. The available dengue notification data do not have information on travel history or clustering of the reported cases. Here, evidence of transmission is indirectly obtained from the notification data via the calculation of the effective reproductive number, *R*_*t*_. This index is interpreted as the average number of secondary cases generated by a primary case at time *t* [34], calculated as the ratio of secondary to primary cases. A value of *R*_*t*_ > 1 indicates a sustained growth of incidence. Since no information is available on who infected whom, the primary cases are inferred from the generation interval of dengue, that is, primary cases are those that could be the source of transmission for the current cases given the time between the onset of their disease symptoms. This approach formally described by Wallinga & Lipsitch [34] results in the following expression for *R*_*t*_:

$$\begin{array}{cc} R_{t} & =\frac{b(t)}{\sum_{a=0}^{\infty }b(t-a)g(a)}, \end{array}$$ (4)

where *b*(*t*) is the number of new cases reported at week *t* and *g*(*a*) is the probability distribution of the dengue generation interval (time between onset of symptoms in a primary case and onset of symptoms in a secondary case) taken as a Normal distribution *N* (*μ* = 3weeks, *σ* = 1week). Confidence intervals for the ratio of two Poisson counts were calculated using the method described in [35] and *R*_*t*_ > 1 was ascertained if *p*(*R*_*t*_ > 1) > 0.95.

The effective reproductive number *R*_*t*_ was calculated weekly for each city, from 2000 to 2015. For each city, an epidemiological year was classified as epidemic if *R*_*t*_ > 1 for at least three consecutive weeks. A period of 3 weeks was chosen because it represents one generation of dengue transmission. We further defined *T*_3_ as the time in weeks from July 2000 to the first observation of three consecutive weeks with *R*_*t*_ > 1 in that city.

### Assessing how vulnerability to dengue importation changed from 2000 to 2015 at the Acrean municipalities in response to changes in the transportation network

In 2001, Acre witnessed its first dengue epidemic, in Rio Branco. Since then, dengue cases have been recorded almost continuously and with great intensity in this municipality. Here we investigate if the spread of dengue from Rio Branco to other municipalities in Acre was associated with the transportation network. First, we investigate if more central cities were invaded earlier than peripheral ones, where centrality is defined as a property of the transportation network (defined below). Secondly, we assessed whether cities closer to Rio Branco were invaded before distant ones.

A structural transportation network was constructed as a graph with 22 nodes representing the municipalities and 29 edges representing the connections between them by (paved, unpaved or under maintenance) roads, waterways or airways, as described in the data subsection. In this network, the edges were considered as unweighted for most of the centrality metrics defined below. The only exception made was for the distance in kilometers from Rio Branco, in which case each edge received a weight equal to the corresponding distance between the connected nodes. We also constructed a mobility network with the same 22 nodes but with a total of 87 edges. As described in the mobility data subsection, in this network the edges are given weights equal to the sum of residents moving from one municipality to another for work and/or study in both directions.

There are several measures of node centrality, the literature being rich with proposals for both weighted and unweighted networks [36]. Each of those proposals focuses on a different aspect of information flow and node properties, therefore producing potentially different node ranks for each measure [37, 38]. Here, we considered the following centrality measures that provide different interpretations in relation to dengue exposure:

- *Betweenness centrality* (*B* and *Bw*): in both unweighted and weighted networks, a node with high betweenness centrality is expected to have a stronger role in the dissemination of information in the network, acting as a bridge between different nodes that are not directly connected. Therefore, they should have higher exposure to infectious diseases and likely contribute to its dissemination in the network. This centrality measure is usually more relevant in large, sparse networks, where those nodes act as bridges between different regions of the network. As defined in [39], betweenness centrality measures the relevance of a node regarding how many shortest paths in the network include that node. We computed an unweighted betweenness measurement (*B*) for each node based on the unweighted shortest path, that is, only based on the vicinity of cities in the structural transportation network. The unweighted shortest path distance between any pair of nodes is the minimum number of edges forming a connected path between them. In principle, each pair of nodes can have more than one shortest path, that is, more than one way of going from the origin node to the destination one with the same number of connected edges. So, if *s*_*m*,*l*_ is the total number of shortest paths from node *m* to node *l*, and *s*_*m*,*l*_(*i*) is the number of those paths containing node *i*, then the unweighted betweenness centrality of node *i*, *B*(*i*), is given by:
  $$\begin{array}{cc} B(i) & =\sum_{m,l}\frac{s_{m,l}\left(i\right)}{s_{m,l}}. \end{array}$$ (5)
  We also computed a weighted betweenness centrality (*Bw*) using the concept of *effective distance* [11] to calculate the shortest path between pairs of nodes in the mobility network. Using this definition, the shortest path between any pair is related to the conditional probability of individuals moving through a particular path between that pair. As defined in [11], the effective distance between any pair of nodes can be obtained through the following steps:
  First of all, for each connected pair of nodes *i* and *j*, with number of travelers from *i* to *j* given by *w*_*ij*_, define the conditional probability of moving from *i* to *j*, given that a person moves from *i*, as
  $$\begin{array}{cc} p_{i,j} & =\frac{w_{ij}}{\sum_{m}w_{im}}. \end{array}$$ (6)
  From that probability, we define the effective distance between connected nodes *i* and *j* as
  $$\begin{array}{cc} d_{eff}\left(i,j\right) & =(1-\text{log}\;(p_{ij})). \end{array}$$ (7)
  From this definition of distance, the length of an ordered path, $\Lambda_{k_{0},k_{1},\ldots ,k_{n}}$, between any pair of nodes *k*_0_ = *m* and *k*_*n*_ = *l*, comprised of *n* edges, is the sum of the effective distance over each connected pair *i*,*j* on that path:
  $$\begin{array}{cc} \Lambda_{k_{0},k_{1},\ldots ,k_{n}} & =n-\text{log}\prod_{i=0}^{n-1}p_{k_{i}k_{i+1}}. \end{array}$$ (8)
  The shortest path in effective distance between nodes *m* and *l* is then defined as that with the shortest length Λ_*m*,…,*l*_. That is,
  $$\begin{array}{cc} s_{eff,m,l} & =\text{min}(\Lambda_{m,\ldots ,l}). \end{array}$$ (9)
  Finally, the weighted betweenness centrality *Bw*(*i*) is computed in the same way as in Eq 5, where the shortest path is obtained from the effective distance definition as in Eq 9.
- *Closeness centrality (C and Cw)*: measures how fast information can flow from a node to all other nodes in the network [37, 38, 40]. In our context, it translates into how fast the pathogen can reach all nodes from a single source based on human mobility. In the case of the unweighted transportation network, for each node *i*, *C*(*i*) was calculated as the inverse of the sum over the unweighted shortest path distance between node *i* and all other nodes in the network. The unweighted shortest path distance *d*(*i*, *j*) from node *i* to *j* is simply the minimum number of edges forming a path along connected nodes between *i* and *j*.
  The closeness centrality *C*(*i*) is given by
  $$\begin{array}{cc} C(i) & =\frac{1}{\sum_{j}d\left(i,j\right)}. \end{array}$$ (10)
  For the mobility network, we calculated the weighted closeness centrality *Cw*. We used the approach by [38] where the weighted distance between any pair of connected nodes is given by the inverse of the number of travelers in the corresponding edge. This is equivalent to redefining the edges’ weight as the inverse of the number of passengers between the given nodes. Therefore, the higher the number of travelers in a given edge, the shorter the distance between them. The weighted shortest path distance, *d*_*w*_(*i*, *j*), is then the connected path with the lowest sum over the weighted distance between each pair on that path. Finally, the weighted centrality of node *i*, *Cw*(*i*) is the inverse of the sum over *d*_*w*_(*i*, *j*) from *i* to every other node in the network, analogous to Eq 10. Therefore, the higher the number of passengers in the shortest paths from a node, the higher its closeness centrality.
- *Strength centrality (Sw)*: this measure is the weighted analog of degree centrality, which is simply the node degree. For weighted networks, strength centrality measures the intensity of the connections of a node, not only their number [41]. Here, *Sw* was calculated as the total number of people regularly commuting to a given city for work or study. The higher a node’s strength, the more it shares individuals with neighboring nodes, potentially increasing its exposure to infected agents present in its network vicinity.
- *Eigenvector centrality (Ew)*: as defined by Bonacich [42], the centrality of a node is defined by the centrality of its neighbors. Therefore, the centrality of a node can be measured by the combination of its connectivity and that of its neighbors. In this sense, the more connections a node have with highly connected nodes, the more likely it is to be exposed to infected agents from a random source. In the case of weighted networks, a node will have high eigenvector centrality (*Ew*) if it has strong connections with highly connected nodes taking into account the weight of those edges. The weight used for this centrality measure is the number of travelers between nodes.
  From the weighted adjacency matrix *W*, the eigenvector centrality *Ew*(*i*) of each node *i* is given by the *i*^th^ entry of the eigenvector corresponding to the largest eigenvalue of matrix *W*.
- *Network Distance from Rio Branco (D, Dw)*: Rio Branco is the municipality with the strongest communication with the rest of the country. The closest (farthest) a node is to Rio Branco, the faster (slower) should be for infection from outside the state of Acre to reach this node once it is introduced in Rio Branco [43]. From structural transportation network, we computed the *distance in degrees from Rio Branco (D)* to a given node *i* as the unweighted shortest path distance from Rio Branco to that municipality. For the mobility network, we computed the *effective distance from Rio Branco, Dw* to each node *i*, which relates distance with the probability of individuals flowing from Rio Branco to the target node in 2010. For the calculations of *D*, two edges were disregarded: the waterway connections between RB-Xpr (Rio Branco-Xapuri) and Xpr-Brl (Xapuri-Brasiléia), because they have weak traffic [44] in comparison to the roadway route.
- *Distance in kilometers from Rio Branco (Dk)*: this measure is calculated as the sum of kilometers in each of the edges in the shortest path from Rio Branco to each target node in the structural transportation network. In the case of connections where both terrestrial and pluvial routes were available between a pair of nodes, we used the former since it is the most frequently used.

To access how vulnerability to dengue importation changed in the study time at the Acrean municipalities in response to changes in the transportation network, we measured, using Spearman correlations, the intensity of the relationship between *T*_3_ to the network descriptors and compare to the structural state of the transportation network during the period of study. While the descriptors for the mobility network are static since we only have the flow of individuals for the year of 2010, the analysis of the transportation network status is dynamic since there were considerable interventions such as construction and paving of roadways during the period of study. We hypothesize that *T*_3_ and the descriptors should be correlated since the road changes in the Acrean network is very evident, and the invasion of dengue in the state occurred first in the nearest municipalities in terms of distance and with better road structure.

The analysis were performed in software R 3.3.2 (R Core Team, 2016, cran.r-project.org/) and in Python 3.5 (Python Software Foundation, www.python.org) using the libraries Networkx [45], Pandas [46], Numpy [47], and igraph [48].

### Receptivity of the Acrean cities to dengue transmission

Receptivity depends on the presence, abundance and vectorial capacity of the local population of *Ae. aegypti*. Moreover, dengue receptivity has been linked to unplanned urbanization, fast population growth, poor infrastructure as lack of urban services and effective mosquito control, and globalization [49]. Here, we review the available information on these topics in order to provide the best description possible for the evolution of dengue receptivity in the study period. First, we investigated the available records of *Ae. aegypti* presence in the region using LI/LIRAa. From this data, we computed the year when *Ae. aegypti* was first recorded in each municipality.

Secondly, we computed how much each municipality changed from 2000 to 2010, in terms of population growth, urbanization, garbage collection, water supply and sanitary sewage.

We also reviewed the scientific literature searching any biological information regarding the vector populations found in Acre. The literature is scarce, and a single study was found on the competence of Acrean *Ae. aegypti* to DENV-2 [50].

## Results

### Vulnerability to dengue importation to Acre

**Fig 2:**
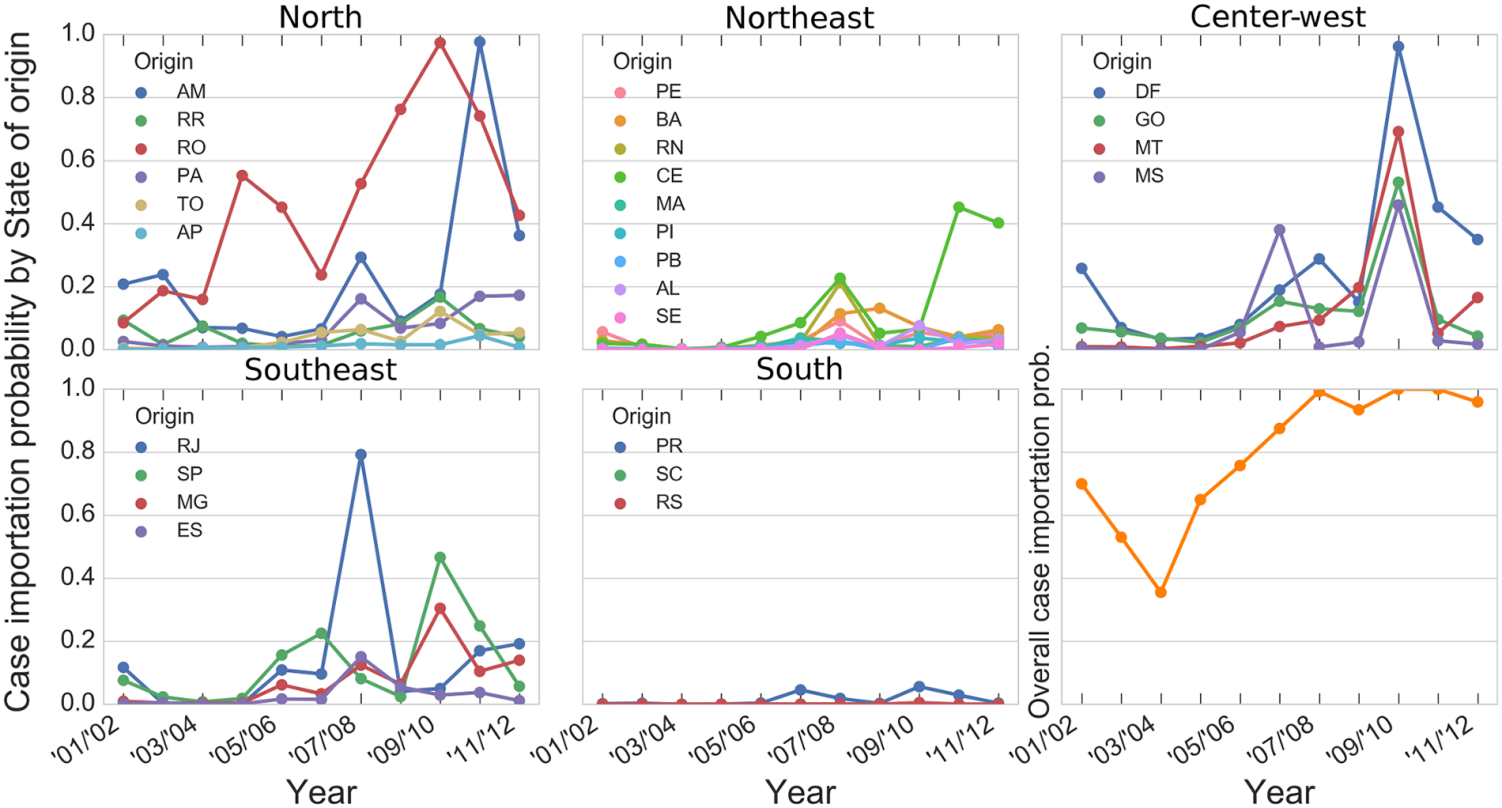
Probability of at least one dengue-infected individual traveling to Acre by state of origin during his/her infectious period, per year. Each panel refers to states within each Brazilian geopolitical Region. The panel on the bottom right shows the overall probability of at least one case importation, regardless of state of origin, as in Eq 3.

From 2001 to 2012, the overall flow of passengers to Acre increased from about 50,000 to more than 150,000 passengers per year. During this period, the main states of origin for travelers to Acre were Distrito Federal (DF), Rondônia (RO), Amazonas (AM) and São Paulo (SP) (Fig 1). Meanwhile, the states of Rio de Janeiro (RJ), São Paulo, Minas Gerais (MG) and Bahia (BA) had the highest dengue cases recorded. As discussed in the methods section, the probability of case importation to Acre is a combination of both the number of travelers and dengue activity. Fig 2 shows the probability of dengue case importation into Acre from 2001/2002 to 2011/2012. It reached ca. 70% in 2001/2002, reducing to its lowest level (38%) in 2003/2004, steadily increasing to ca. 100% from 2004/2005 to 2007/2008, remaining so until the end of the study period. The north region was found as the main source of dengue cases to Acre, in particular, the neighboring state of Rondônia (RO), followed by the center-west (Distrito Federal-DF and Mato Grosso-MT), southeast (Rio de Janeiro-RJ and São Paulo-SP), and northeast (Ceará-CE). The contribution of Rondônia, Distrito Federal and Mato Grosso to the exportation of dengue to Acre was mainly associated with the flow intensity between these states while that of Rio de Janeiro and São Paulo was a result of the combination of moderate flow intensity and high rates of dengue activity. Others states such as Tocantins (TO), Roraima (RR) and Amapá (AP) in the north, Maranhão (MA), Piauí (PI), Paraíba (PB) and Alagoas (AL) in the east and all states in the south region were unlikely exporters of dengue cases to Acre due to the combination of low flow and low dengue activity.

### Dengue establishment in the Acrean municipalities

The first autochthonous dengue case was reported in Acre in 2000. From this year until 2008, the annual incidence did not exceed 900 cases per 100,000 inhabitants, a level of activity similar to those found in the other northern states of Brazil, except for Roraima. However, in 2009 there was a significant increase in dengue incidence in Acre when it tripled to an alarming level of 2,800 cases per 100,000. In 2010, dengue incidence was even higher: 4,793.3 cases per 100,000 [51]. According to the Ministry of Health, in 2011, Acre was among the states classified as at moderate risk, and within the state, the capital Rio Branco was classified as at the highest risk [52]. On the other hand, the Alto Juruá region, in the northwest of Acre, which until 2014 had not yet registered autochthonous cases of dengue, witnessed its first dengue epidemic in Cruzeiro do Sul. In 2014, Cruzeiro do Sul was among the municipalities with the highest number of dengue cases between the epidemiological weeks 01 and 47 of 2014. Compared to 2013, the number of cases jumped from 30 cases to 23,130 [53, 54].

**Fig 3:**
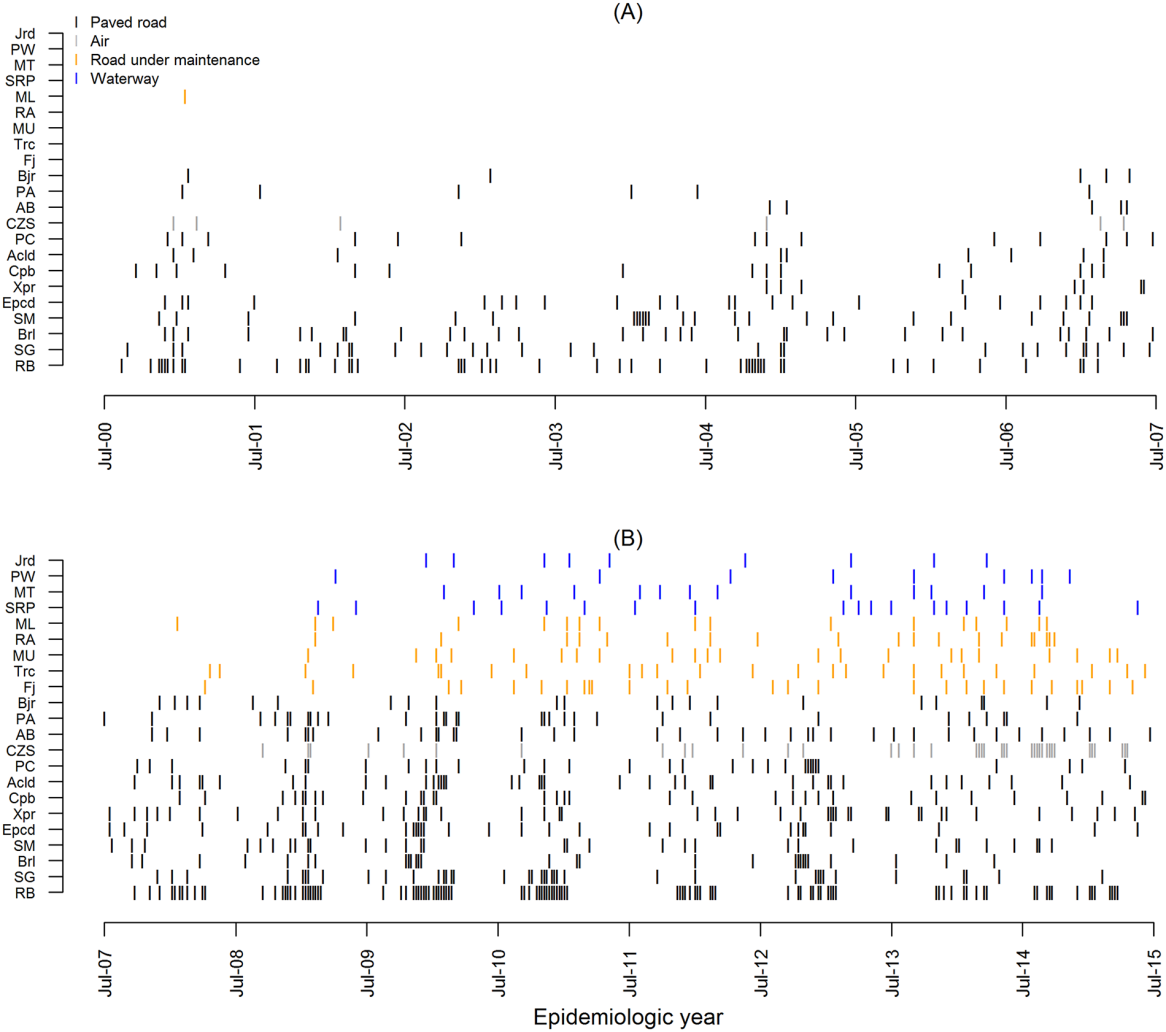
Weeks with *R*_*t*_ > 1 from July 2000 to July 2015. (A) Weeks with *R*_*t*_ > 1 from July 1^st^, 2000, to June 30^th^, 2007. (B) Weeks with *R*_*t*_ > 1 from July 1^st^, 2007, to June 30^th^, 2015. Each line on the vertical axis corresponds to a municipality in the state of Acre. Black: municipalities accessible by paved roads during all or almost all the time of the study. Gray: city (CZS) served by airline from Rio Branco. Orange: municipalities partially or totally accessible via unpaved or under maintenance roads. Blue: municipalities whose access necessarily include waterways. Refer to Table 2 for municipalities’ name.

In Fig 3 we show the weeks with *R*_*t*_ > 1, from 2000 to 2015 at each Acrean municipality. A sequence of weeks with *R*_*t*_ > 1 is an indication of epidemic growth. All municipalities presented this event at least once during the study period. The municipalities that presented the lowest percentages of weeks with *R*_*t*_ > 1 were Porto Walter (PW) and Jordão (Jrd) (1.15% for both) throughout the study period. These are the two most peripheral in the state, and whose access includes a stretch traveled by river. The ones with the highest percentages of “epidemic weeks” were Rio Branco (RB, 9.32%), Senador Guiomard (SG, 6.39%), Epitaciolândia (Epcd), and Brasiléia (Brl) (6.00% for both).

In Fig 3, colors indicate the main type of access to each city. There are two main types of access, those cities in which access is by road (black and orange) and those in which the final access is by waterway (blue). Before 2008/2009, only cities in which access is by road registered *R*_*t*_ > 1. However, these records were mostly intermittent, as can be seen for example in Bujari (Bjr), Capixaba (Cpb), Assis Brasil (AB), Porto Acre (PA), Acrelândia (Acld), Plácido de Castro (PC) and Xapuri (Xpr). Cruzeiro do Sul (CZS) in gray, which is the only city served by an airline to Rio Branco, also registered few weeks with *R*_*t*_ > 1 before 2008. After this year, when several paving works and road maintenance were concluded, the frequency increased eventually becoming more intense culminating with CZS recording the first sustained epidemic in 2014. The time frame of infrastructural changes and its potential impact on dengue cases notification is discussed in more detail in the section Dengue spread in Acre. The municipalities in orange, Mâncio Lima (ML), Rodrigues Alves (RA), Tarauacá (Trc) and Manoel Urbano (MU), also have access by road, but part of the final access was under construction or maintenance during all or almost all the period of study. In these municipalities, as well as in those with final access by waterway, the first records of *R*_*t*_ > 1 occurred in the beginning of 2008, in a slow way, while in those cities with access by waterway, the records were even rarer than those accessible by roads.

### Vulnerability to dengue importation within Acre

Public investments since 2000 in the development of the Acrean transportation infrastructure, including the construction of the Pacific Highway and the pavement of intermunicipal roads, have brought more connectivity between the municipalities, in particular within southeast Acre, composed by Rio Branco, Senador Guiomard, Capixaba, Xapuri, Epitaciolândia, Plácido de Castro, Brasiléia, and Acrelândia. In contrast, transportation and mobility between the southeast and other regions of Acre (Alto Juruá) is still very difficult due to road conditions, although improvements have occurred during this period. The accessibility to the northwest area of Acre began to improve after the year 2008, when paving of the highway BR-364 in the region of Manuel Urbano (MU) and Feijó (Fj), in the central area of Acre. Highway BR-364 is the only one that crosses the state from Acrelândia (Acl, in the southeast) to Mâncio Lima (ML, in the northwest) and connects Acre to the rest of Brazil via Rondônia state.

Highway BR-364 has been under construction since the beginning of the last decade. In southeast Acre the paving and restoration of this highway were completed quickly, and only the stretch from Acrelândia to Rio Branco was still under construction until 2007/2008. The stretch from the capital to Cruzeiro do Sul, at the northwest part of the state, passes through very unstable soil, making it difficult to maintain during the monsoon season. Only in 2012/2013, this stretch was completely paved, but in the following years repairment works were needed. Besides this highway, an extremely important link between the southeast and northwest Acre is the daily flight between Rio Branco and Cruzeiro do Sul, which increased by 18% from 2001 to 2012. The most remote municipalities are Santa Rosa do Purus, Jordão, Marechal Thaumaturgo and Porto Walter with very limited access and only by river, most of them only by small and medium-sized vessels depending on the time of year. On the other hand, air travel alone was responsible for an average of 134 passengers to Acre per day during the epidemiological year 2001/2002, steadily increasing to almost 500 per day in 2013/2014, most of them to Rio Branco, responsible for approximately 90% of all air travel to Acre from other Brazilian states. To illustrate the impact of interstate airflow in this population, the 2010 Census registered 3.6 thousand commuters to Rio Branco, so that the average daily out-of-state air passengers corresponded to almost 4% of daily commuters in 2001/2002, up to almost 14% in that of 2013/2014. This data contrasts with the common misconception regarding air travel to Acre and its impact to information inflow, which is believed to be negligible due to its relatively low volume with respect to the rest of the Country.

**Fig 4:**
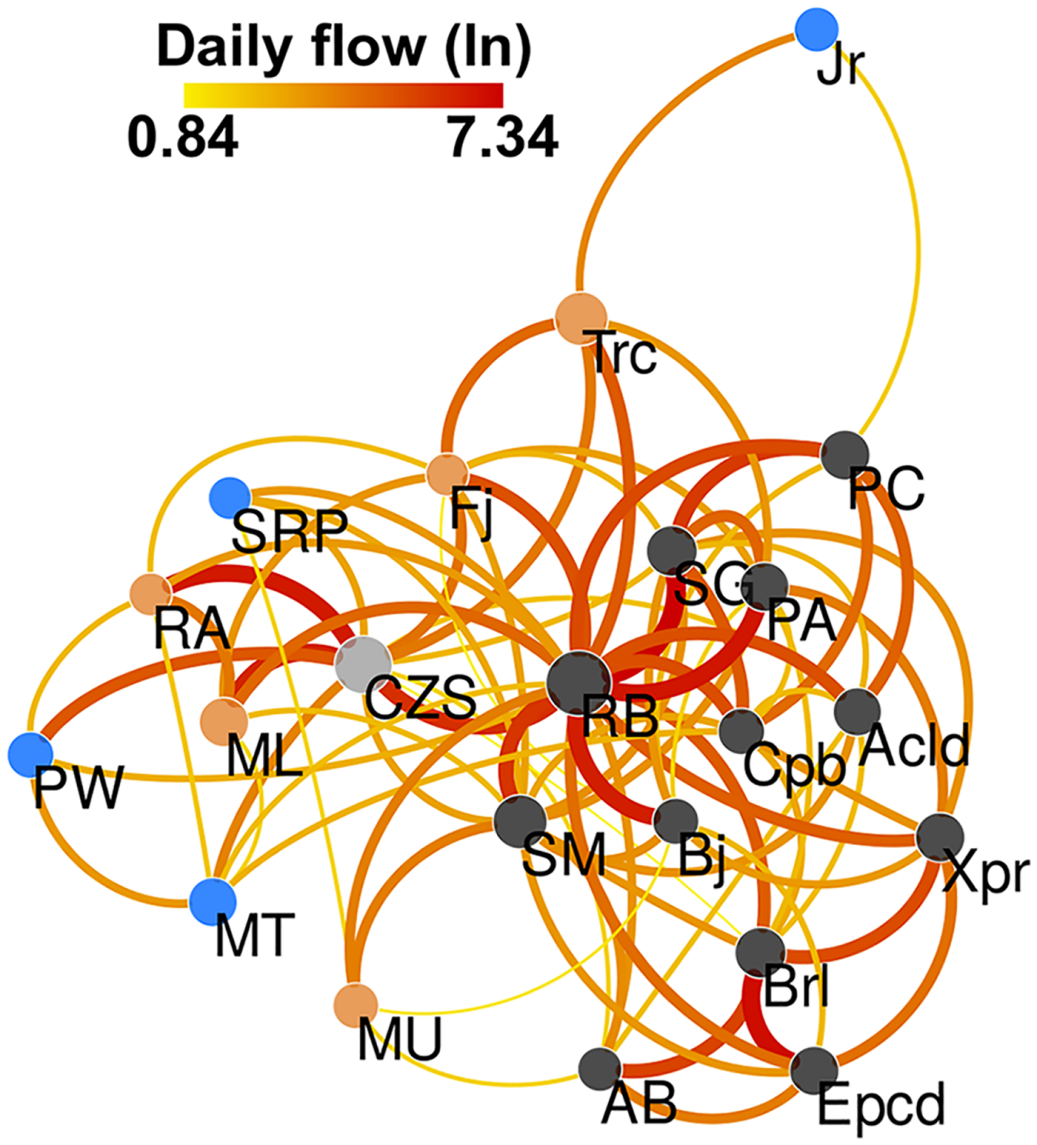
Mobility network between Acrean municipalities. Each node represents a municipality, with its size proportional to the natural logarithm of the population. Edges represent the average flow of individuals between municipalities, with width and color proportional to the natural logarithm of the number of travelers. Node color follows the same criteria as in Fig 3: (black) municipalities connected by paved roads, (gray) municipality with air connection to RB, (orange) municipalities connected by highway BR-364 with unpaved or maintenance sections during the period of study, (blue) municipalities that are mainly accessed by river.

The connectivity between municipalities is shown in Fig 4. We used Cytoscape software [55] to build a representation of the Acrean municipalities’ mobility network, with edge width and color proportional to the natural logarithm of the daily number of travelers and node size proportional to the natural logarithm of each municipality’s population (node color indicate access mode, as in Fig 3). Nodes position are defined based on the Fruchterman–Reingold algorithm [56], which is a force-directed layout widely used in network visualization. In this layout, all nodes have a repulsion force between them, while edges weight are used as an attractive force between connected nodes. Nodes that share stronger connections, i.e., with more travelers, are placed closer to each other than those that share weaker or no connection. Therefore, groups of nodes sharing stronger bonds among them form regional clusters in this abstract space of connectivity. This particular visualization facilitates the detection of municipalities that share relatively strong bonds even when geographically far apart. On the other hand, nodes that are placed on the periphery with this algorithm are those that have relatively few and/or weak connections. We can see that the two most populous municipalities, the capital Rio Branco (RB) and Cruzeiro do Sul (CZS), are important hubs in this network. The access mode (waterway, paved and unpaved roads, airway) confers a natural clustering on the mobility network, suggesting an intuitive relationship between ease of access (transportation network) and the number of travelers (mobility network). Municipalities accessed by paved roads are more densely connected with each other, while the strong connection between Rio Branco and Cruzeiro do Sul, which are connected by airports, considerably shortens the effective distance between east and west side of the state. Another example of this relationship is the fact that municipalities which are mainly accessed via waterways are peripheral in the mobility network. In fact, Marechal Thaumaturgo (MT), Jordão (Jrd) and Santa Rosa dos Purus (SRP) have the lowest *Ew*, *Cw* and *Sw* centralities, with Porto Walter (PW) also among the five municipalities with lowest values for those centralities.

The capital Rio Branco is the most central node in the Acrean network with respect to all descriptors used (see in S2 Appendix) being the most present node in all shortest paths (as measured by *B* and *Bw*); the municipality with the smallest average shortest path to all other nodes (*C* and *Cw*); the strongest attractor (*Ew*) in the network; and the node with most travelers (*Sw*). Therefore, it is both the most vulnerable to exposure from a pathogen present in the state at a random municipality, as well as the node which poses the highest impact in other municipalities’ vulnerability once infected. Porto Acre, Bujari and Senador Guiomard, all with geographical borders with Rio Branco, also present high centrality measures, therefore sharing similar relevance to disease transmission. All of those cities are located on the east side of the state, and all of them can be accessed via paved federal highway.

Another municipality with high centrality for most indicators is Cruzeiro do Sul (CZS), on the west side of Acre. Although geographically distant from Rio Branco, this municipality shares an airport connection with the capital, drawing a significant amount of travelers and therefore shortening its network distance with respect to individual flow. Being the second most populous municipality, it also shares strong connectivity with its geographical neighbors. In fact, it has the third highest strength centrality, that is, the third municipality with highest number of travelers. Due to its connectivity to Rio Branco and its role as western hub, Cruzeiro do Sul acts as an important bridge between the capital and the western region.

### Dengue spread in Acre

**Fig 5:**
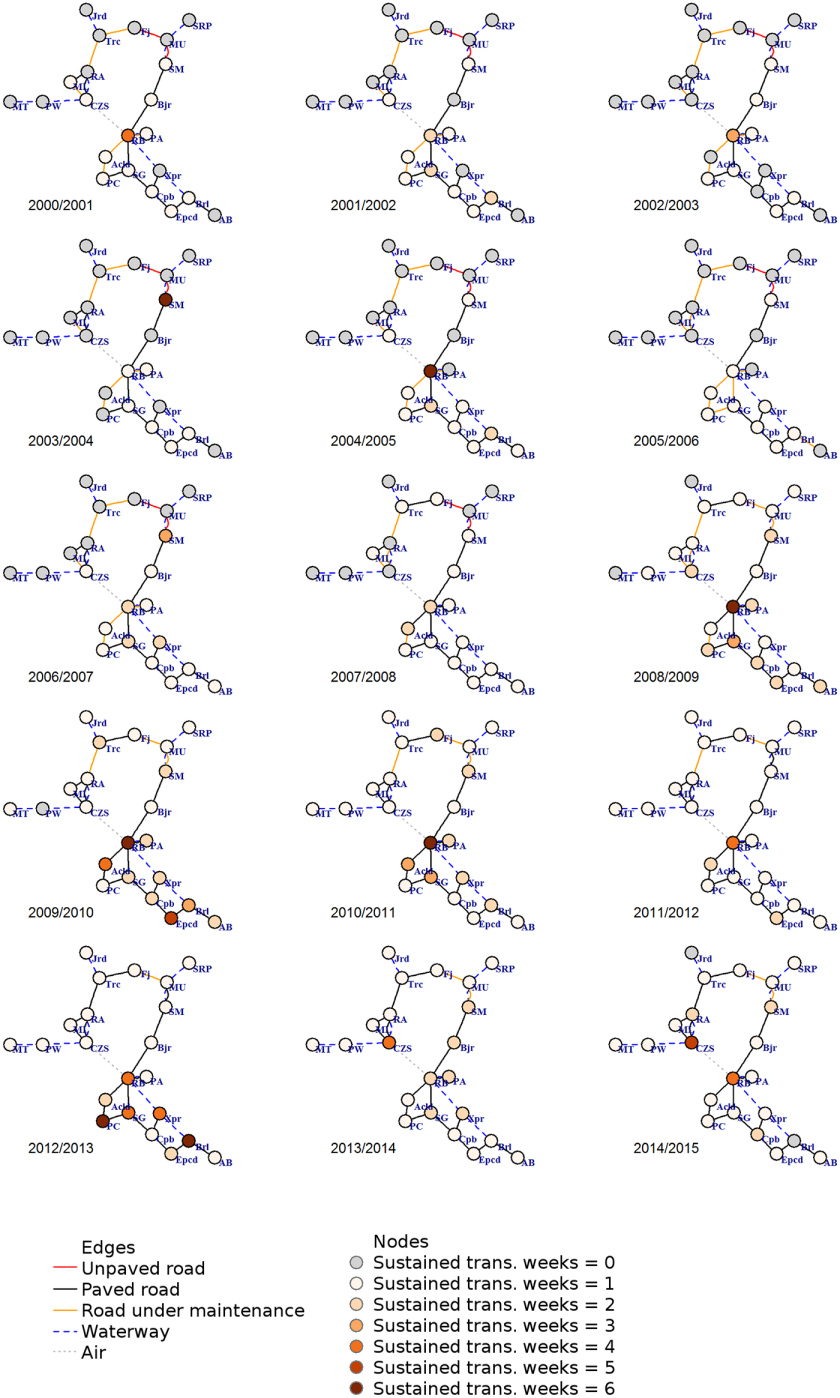
Structural networks time series in epidemiologic years. Nodes (circles) are the 22 municipalities of the state of Acre in which gray nodes are municipalities with *R*_*t*_ < 1. Colored nodes are the representations of *R*_*t*_ > 1 by the number of consecutive weeks per year, varying in shades of orange for at least one week (lighter) to six consecutive weeks (darker), with gray color for years with no such event in any week. Edges (lines) are the direct connections between municipalities by modal type category: roads (unpaved in red, paved in black, under maintenance or construction in orange), waterway in dark blue dashed line and air in dotted gray line. This figure is provided in higher resolution in supplementary information S1 Fig.

In Fig 5 we can observe a sequence of panels showing how dengue invaded and established itself in Acre, following the transportation network. The nodes represent the cities, colored according to the number of consecutive weeks with *R*_*t*_ > 1 per year. In this study, we consider “at least three consecutive weeks with *R*_*t*_ > 1” as a marker of (at least temporary) disease establishment. Of the 22 Acre counties, nine were positive for this marker at least once during the study period despite the fact that all of them registered at least one case of dengue during this time. Until 2008/2009, only Rio Branco e Sena Madureira had evidence of disease establishment according to this criterion. The two cities are well connected in terms of human mobility, close regarding geographical distance, and have paved roads connecting them. At the end of the study period, other seven cities presented at least three consecutive weeks with *R*_*t*_ > 1. Of those, six are in southeast Acre, in the most connected and best-accessed region. The other city is Cruzeiro do Sul, one of the most distant cities in kilometers from Rio Branco, but populous and with an air connection to the capital.

**Fig 6:**
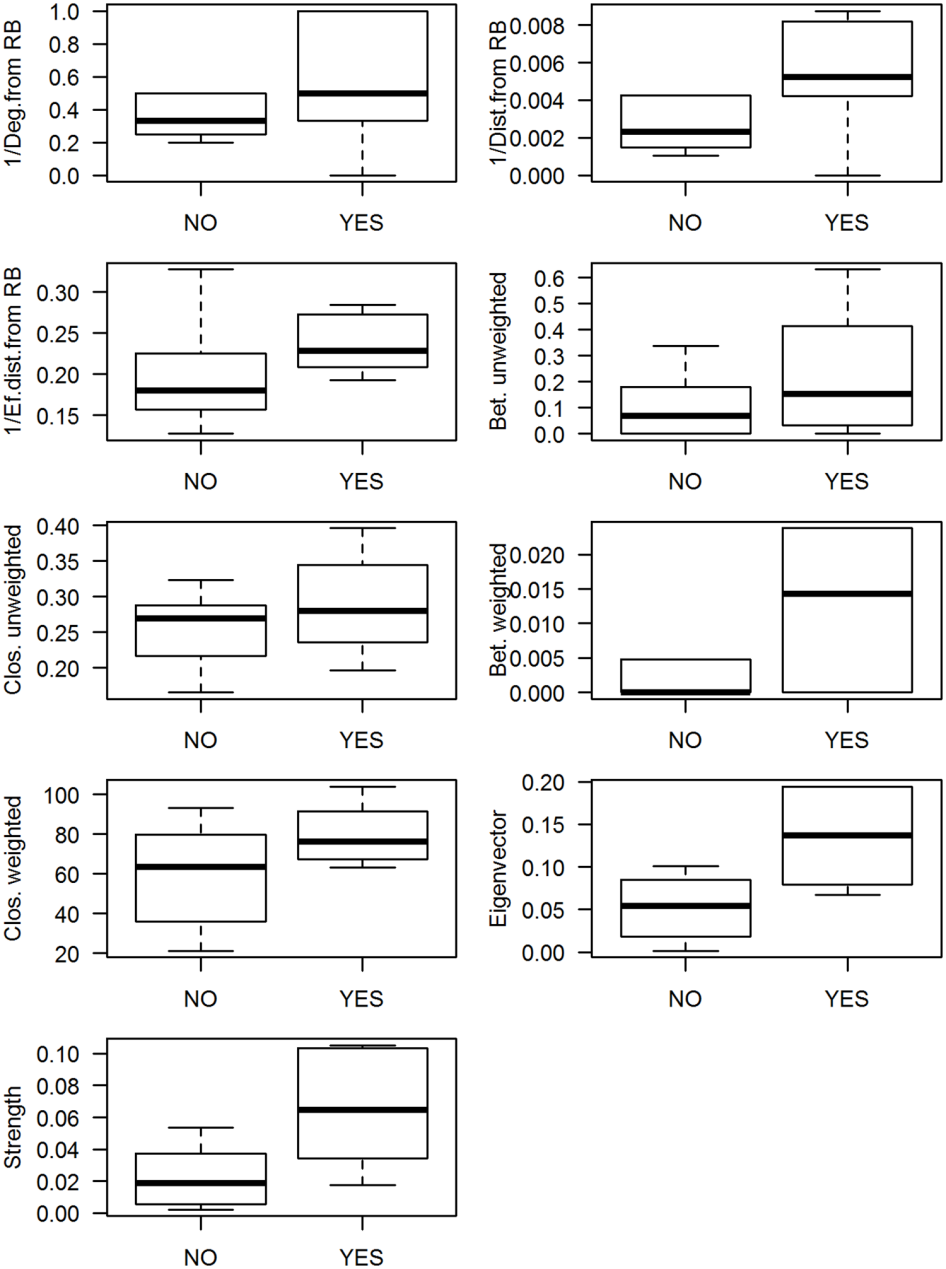
Centrality measures and dengue establishment. Boxplots comparing the network descriptors of municipalities that witnessed dengue establishment during the study period and those which did not (Yes/No). Values for each Municipality are available in S2 Appendix.

We further investigate if there is evidence of correlation between the centrality of a municipality and the time it took for dengue to establish itself, measured by *T*_3_, as defined previously. A summary of the main results is shown in Table 1. This is an exploratory analysis only, since the sample size is small for modeling. Strength centrality was the network descriptor most negatively correlated with *T*_3_. This result suggests that municipalities in which the sharing of individuals with other municipalities was larger had a greater exposure to dengue and, consequently, smaller *T*_3_ than those with lower integration. The Eigenvector centrality of the municipalities, which is related to how strong is the connectivity of a node’s neighborhood, therefore its ability to concentrate population flow in the network, also presents negative correlation with *T*_3_. This is an intuitive result since this property enhances the probability of a node being invaded once a node in its vicinity presents an outbreak, even when not in direct contact. Finally, the distance from Rio Branco, measured by both the effective distance and in kilometers, also showed high correlation with *T*_3_. It is interesting to note that, geographical distance aside, topological properties of the unweighted structural network are not significantly correlated with *T*_3_. It is important to highlight that about half of the Acrean municipalities did not meet the criteria of dengue establishment, which has a significant impact on the correlation obtained. With that in mind, we also investigated if those network properties were correlated with the event of dengue establishment itself. In Fig 6, we show boxplots comparing the centrality of municipalities that witnessed dengue establishment during the study period and those which did not. This comparison allows for the evaluation of whether municipalities grouped by occurrence (YES/NO) are also characterized by significantly distinct network properties. It is possible to identify that the descriptors most correlated to *T*_3_ were also those that differed the most between municipalities with and without dengue establishment, which are the geographical and effective distance to Rio Branco, Eigenvector and Strength centralities.

**Table 1:** Spearman correlation between time to dengue establishment (*T*_3_) and centrality indicators.

	Indicators	Spearman (*ρ*)	p-value
Unweighted structural network	1/Distance in degrees	-0.36	0.0972
	1/Distance in kilometers	**-0.51**	0.0159
	Betweenness	-0.38	0.0773
	Closeness	-0.27	0.2220
Weighted mobility network	1/Effective distance	**-0.52**	0.0124
	Betweenness	-0.47	0.0631
	Closeness	-0.45	0.0353
	Eigenvector	**-0.52**	0.0125
	Strength	**-0.58**	0.0043

### Receptivity of the Acrean municipalities to dengue transmission

The first record of *Ae. aegypti* in Acre occurred in 1995 in Rio Branco [57] (Table 2). Gradually the nearest municipalities in the southeast of the state also started recording it, and in 2001, eight municipalities had confirmed the presence of *Ae. aegypti*. These municipalities are the same municipalities where *R*_*t*_ > 1 was detected earlier in time, being municipalities along BR-317 (SG, Cpb, Xpr, Epcd and Brl) or with a direct connection to RB (PA) and two with good access to BR-364 since the beginning of the study (SM and Acld). From 2002 to 2006, another three municipalities reported the presence of *Ae. aegypti*: Plácido de Castro, Bujari and Assis Brasil. Bujari is the nearest neighbor of Rio Branco, but *Ae. aegypti* presence was not confirmed until 2006. It is difficult to ascertain the specific causes for this. On the one hand, this was a predominantly rural municipality in the early 2000’s. On the other hand, there was not a well-structured vector surveillance program implemented in small towns such as Bujari until later. Plácido de Castro and Assis Brasil are frontier municipalities, the former at the border with Rondônia state, receiving a considerable traffic of vehicles from the highly dengue endemic neighbor state. It is reasonable to infer that *Ae. aegypti* could be present there previously but unnoticed. However, dengue only established itself in Plácido de Castro in 2012/2013, despite the consistent detection of cases since 2001. Assis Brasil is a small town at the triple frontier Brasil-Peru-Bolivia. In 2000, it had a considerably small population, which may have contributed to the delayed invasion of *Ae. aegypti*.

**Table 2:** Population size and urban/rural ratio in 2000 and 2010, municipalities with *R*_*t*_ > 1 for 3 consecutive weeks for epidemiological years and first year of *Aedes* presence confirmed by LI/LIR*Aa* for 22 Acrean municipalities.

Municipalities	Abbrev.	Population 2000	Population 2010 (% increase 2000-2010)	Urban/Rural ratio 2000	Urban/Rural ratio 2010	Garbage collec. 2010 (%)	Water sup. 2010 (%)	Sanitary sewage 2010 (%)	Epidemiological years with *T*_3_	First year of *Ae. aegypti* confirmed by LI/LIRAa
Rio Branco	RB	252885	336038 (32.88)	8.45	11.22	93	53	58	Almost every year	1996
Senador Guiomard	SG	19766	20179 (2.09)	0.78	1.70	64.2	34	24.1	2008/2009, 2010/2011, 2012/2013	2000
Brasiléia	Brl	17013	21398 (25.77)	1.13	2.00	67.9	55.8	29.6	2009/2010, 2012/2013	2000
Sena Madureira	SM	29412	38029 (29.30)	1.22	1.94	63.3	38.6	13.3	2003/2004, 2006/2007	2001
Epitaciolândia	Epcd	11019	15100 (37.04)	2.04	2.37	68.6	60.4	21.2	2009/2010	1999
Xapuri	Xpr	11952	16091 (34.63)	1.00	1.79	64.2	51.4	29.3	2012/2013	2000
Capixaba	Cpb	5206	8798 (69.00)	0.41	0.81	44.2	39.1	34	-	2001
Acrelândia	Acld	7934	12538 (58.03)	0.79	0.89	48.8	29	11.7	2009/2010, 2010/2011	-
Plácido de Castro	PC	15161	17209 (13.51)	0.85	1.52	55.4	34.5	14.6	2012/2013	2003
Cruzeiro do Sul	CZS	67371	78507 (16.53)	1.37	2.39	68.3	54.1	13.9	2013/2014, 2014/2015	2008
Assis Brasil	AB	3493	6072 (73.83)	1.61	1.56	61.4	49.7	24.8	-	2005
Porto Acre	PA	11403	14880 (30.49)	0.13	0.15	51.1	24.5	12	-	2001
Bujari	Bjr	5829	8471 (45.33)	0.39	0.77	47	40.1	19.4	-	2006
Feijó	Fj	26733	32412 (21.24)	0.73	1.05	51.3	13.5	10.3	-	2014
Tarauacá	Trc	26022	35590 (36.77)	1.15	1.19	49.2	42.2	10.9	-	2015
Manoel Urbano	MU	6370	7981 (25.29)	1.06	1.95	53	58.1	12.9	-	-
Rodrigues Alves	RA	8097	14389 (77.71)	0.48	0.43	38.5	43.1	9.2	-	-
Mâncio Lima	ML	11074	15206 (37.31)	1.09	1.36	50.9	54	6.7	-	2015
Santa Rosa do Purus	SRP	2247	4691 (108.77)	0.30	0.68	47.2	47.8	4.8	-	-
Marechal Thaumaturgo	MT	8294	14227 (71.53)	0.13	0.39	32.6	23.9	8.4	-	-
Porto Walter	PW	5486	9176 (67.26)	0.36	0.57	20.6	38.5	0.7	-	2015
Jordão	Jrd	4459	6577 (47.50)	0.24	0.53	38.3	36.1	5.3	-	-

LI/LIR*Aa* stands for household larval survey (LI) and Rapid Assessment of Infestation by *Aedes aegypti* (LIR*Aa*); *T*_3_ refers to the occurrence of at least three consecutive weeks with *R*_*t*_ > 1; Epidemiological year for dengue in Brazil was defined as the period from July of base year to June of the next.

Between 2007 and 2013 no other city reported the presence of *Ae. aegypti*. The only exception being Cruzeiro do Sul, in 2008. This invasion was interrupted, however, and the mosquito was detected again only in 2013 [58]. From the municipalities to the north of Sena Madureira along the BR-364 highway, four confirmed the presence of *Ae. aegypti* in 2015: Feijó, Tarauacá, Mâncio Lima and Porto Walter. The first three, together with Cruzeiro do Sul, are located along the BR-364 and are important stopping points for those traveling. For the same reason, it is not possible to affirm that the mosquito was actually absent before that, since monitoring was not implemented. Five municipalities remained *Ae. aegypti* free as of 2015, according to the LI/LIRAa dataset (Table 2). These are the most peripheral municipalities of Acre, and also the most rural.

Structural characteristics of Acrean municipalities can be part of the explanation for the slow invasion and establishment of *Ae. aegypti* in the state. According to IBGE’s urban/rural classification, Acre is mostly a rural state, but the population of all municipalities and the percentage of urbanization increased from 2000 to 2010. The exceptions are two, Rodrigues Alves and Assis Brasil, where total population increased as well but the urban population decreased [25]. Both municipalities presented an increase in the number of rural settlements [59]. Population growth, coupled with the increase in urbanization without improving the coverage of general services—such as garbage collection, water supply and sanitary sewage (Table 2) –, contribute to the fact that, even in a more rural state, the ideal conditions for establishing the mosquito are guaranteed. Those characteristics increase the difficulty in controlling *Ae. aegypti* [50] and consequently the local receptivity.

## Discussion

In the process of describing the introduction of dengue in Acre, we assessed the contribution of network metrics and reproductive numbers as indicators of vulnerability, disease establishment and receptivity in the study of disease emergence. In the landscape epidemiology of infectious diseases, the description of the temporal dynamics of the populations of hosts, vectors, and pathogens, all spatially interacting in a favorable environment, contributes to the understanding of what characteristics and factors favor disease transmission an establishment [60]. Even small differences in landscape composition, which are often unapparent, can alter the microhabitats of the vector and thus the conditions that allow the amplification of a pathogen [60]. In addition, the dispersion of pathogens and vectors is directly linked to the development of transportation networks and increased globalization [49]. In this context, human population expansion has significantly affected the epidemiology of vector-borne diseases, creating a large urban continuum, altering the landscape structure and providing rapid mechanisms for hosts and pathogens to disperse [60].

The results of this study suggest that the landscape changes that occurred in the last decade have created favorable conditions for the establishment of dengue virus transmission, bringing together all the fundamental factors for its occurrence: the man, the virus, the vector, and especially the environmental, political, economic, social and cultural conditions favorable for the establishment of the transmission chain [61]. In Acre, the revitalization of its major roads, as well as the increased accessibility by air both to and within the state, have increased dengue vulnerability. Notice that the increase in the flow of people and importation of dengue cases to Acre coincides with when disease dispersion becomes more pronounced within the state, suggesting a synergy between increasing vulnerability of the state at a global scale and increasing local vulnerability among municipalities, fueling viral spread in the region. Human mobility is responsible for viral spreading, as both asymptomatic humans or those with very mild symptoms continue to carry out their tasks and take the virus to other regions. Humans are also responsible for possibly transporting the mosquito itself (which can be infected or not) by air, road, and waterway [2, 7, 62].

Some network descriptors used in this study were useful for characterizing the level of centrality/periphery of the Acrean municipalities and its relationship with dengue importation. The mobility network that connects the Acrean counties is small and dense—22 nodes and density 0.38 –, so centrality measures such as betweenness (*B* and *Bw*) and closeness (*C* and *Cw*) were not as relevant as they would be in larger and sparser networks. However, the strength (*S*) was the most relevant of the descriptors, because, in a small network, the relationship between local and global characteristics are stronger. Rio Branco is the main attractor of the state, with highest and strongest connectivity within Acre, since most of the routes connecting municipalities in the northwest to the southeast region pass by the capital. Also, it is a reference center for the entire state. A node with high connectivity, that is, a node to which many others connect to and where there are many outputs, is an essential node for the propagation of infectious diseases [63]. Therefore, investing in dengue control and prevention in a systematic manner in Rio Branco has potentially a strong impact throughout the state, not only because of its connectivity within Acre but also because this city is the main port-of-entry from other Brazilian states.

The structure of the network, the distribution of degrees between nodes and the main routes are known to impact the spread of diseases [64]. In fact, we can observe that dengue first spread in the vicinity of Rio Branco and then, after an extended period of time, approximately 13 years, the first event of sustained transmission (here defined as at least three consecutive weeks with *R*_*t*_ > 1) occurred in Cruzeiro do Sul. The flow of people by air between Rio Branco and Cruzeiro do Sul, and the improvement of the BR-364 were very significant predictors of this first epidemic in the northwest region of Acre. Although Cruzeiro do Sul confirmed the presence of the *Ae. aegypti* only in 2008 [58], ever since the beginning of the study there were records of cases in the municipality, even when there were no records in intermediate connections by road between both municipalities. This last result highlights the importance of the airline connection between them for dengue case importation.

The mobility data used in the present work is from the 2010 Census [30] when the structural network around Cruzeiro do Sul and Rio Branco was already stabilized regarding paving and maintenance, as shown in Fig 5. Since dengue epidemiological year of 2009/2010, all roadways directly connected to those two hubs had their maintenance concluded. Also, the unpaved connections to and from Manoel Urbano, which is structurally in between the two Acrean hubs, had also improved by that time. Most of its paving work was concluded by the end of 2010, although maintenance works continued for the rest of the study period. Despite the unavailability of detailed movement for performing a rigorous analysis, it is to be expected that during the period with unpaved and with major maintenance works in the roadways the flow of individuals was lower than when those structural bottlenecks were resolved. Therefore, we expect that the 2010 mobility flux data is more representative of the work/study-related movement from 2009 onwards. Before that, we conjecture that this particular type of human mobility was more concentrated over those connections with paved roads and without major maintenance work.

The structural network status evolution depicted in Fig 5 allows us to identify a few key factors regarding the temporal evolution of ease of access and dengue case notification. Up until epidemiological year 2009/2010, when paved roads (black edges in that network) were mainly present in the vicinity of Rio Branco, the occurrence of *R*_*t*_ > 1 was also mostly present in that area—which can be identified by node’s color in Fig 5 as well as by the markings in Fig 3. In the area surrounding Cruzeiro do Sul, which was mainly connected by waterways (dashed blue edges) and roads under maintenance (orange edges), before 2007/2008 the occurrence of *R*_*t*_ > 1 was registered only once at Mâncio Lima (ML) and a few times in Cruzeiro do Sul. These results reinforce the hypothesis that those cases notified in Cruzeiro do Sul were most likely imported cases from Rio Branco due to the airline connection. It is interesting to note that in terms of roadways, the path from Rio Branco to Cruzeiro do Sul had a structural bottleneck at Manoel Urbano (MU) since it was only accessed by means of unpaved road (red edges) up to 2007/2008.

Improvements in the structural network started in 2008/2009, which included paving and maintenance work of the roadways around Manoel Urbano, and were consolidated in 2009/2010 with the conclusion of major maintenance work in the roadways directly connected to both Rio Branco and Cruzeiro do Sul. We can see that is was precisely after those improvements that dengue notifications started to occur throughout the state of Acre, as well as the number of consecutive weeks with *R*_*t*_ > 1 in each municipality. The combination of low occurrence of *R*_*t*_ > 1 during the years with unpaved and under maintenance roadways, and the increased observation of *R*_*t*_ > 1 after improvements were implemented reinforces the hypothesis that the human mobility network and the structural changes were relevant to the observed geographical spread of the virus in the state of Acre.

As for demographic characteristics, Acrean municipalities are marked by having small populations (Table 2). The capital Rio Branco, the one with the largest population, has 336,038 inhabitants [25]. The second largest municipality in population size is Cruzeiro do Sul, with 78,507. The remaining ones do not exceed 40,000 inhabitants, the majority of them having less than 20,000 inhabitants. The peripheral municipalities according to the network descriptors are also those with the smallest populations and are, mostly, more rural than urban. These municipalities are less vulnerable and less receptive to dengue, which is seen by the low occurrence of *R*_*t*_ > 1 and no event of sustained transmission as defined in this study. Nonetheless, in the world, there has been increased reporting of *Ae. aegypti* invasion and dengue introduction in less urbanized areas. In Colombia, dengue-infected *Ae. aegypti* was found in rural regions [65] south of Bogotá. In Nicaragua, a study showed the unsuspected presence of dengue cases in those areas as well [66].

These results from the literature and the results shown in the present manuscript suggest that introduction and establishment of dengue in those peripheral areas of Acre could be just a matter of time, especially since the virus has already been able to establish itself in the state.

Overall, with this study, we understand that the determination of vulnerable and receptive localities for dengue in the state of Acre is essential for an effective action of entomological and epidemiological surveillance. It is fundamental that all municipalities, especially those with a roadway link, systematically monitor mosquito infestation, which is not currently the case. In addition, favorable conditions for mosquito development are present in Acrean municipalities, with low coverage of services, climate and increased urbanization [25], which probably impacts on the availability of breeding sites for *Ae. aegypti* and its establishment. Acre has a humid equatorial climate, which may contribute to the transmission of the dengue virus occurring throughout the year. According to [50], the mosquitoes of the region have the vectorial competence to transmit the DENV-2. Unfortunately, we did not find more biological information on the mosquito populations of Acre. We can conclude that Acre is a state at risk of major dengue epidemics, with entire populations susceptible to all serotypes of the disease. Due to the limited data and number of municipalities, our analysis is more descriptive/qualitative than quantitative. Nonetheless, we believe that the descriptors discussed here as well as the methodological approach has proved a valuable way to assess invasion risk which can be easily expanded to larger areas—conditioned on data availability –, allowing for a more quantitative analysis..

## Supporting information

S1 Appendix Airline origin-destination flow estimates based on Brazilian airline database. Description of mathematical framework to estimate airline origin-destination flow between Brazilian airports from publicly available data.
(PDF)

S2 Appendix Network descriptors and correlations between them. Full matrix of centrality measures for each Acrean Municipality and confusion matrix of those measures.
(PDF)

S1 Fig High resolution version of the structural network figure. Vectorial format of the structural network of Acrean municipalities for each dengue epidemiological year from 2000/2001 to 2014/2015.
(PDF)
