# Supplementary material for: The introduction of dengue follows transportation infrastructure changes in the state of Acre, Brazil: A network-based analysis
Source: PLoS Negl Trop Dis. 2017 Nov 17;11(11):e0006070. doi: 10.1371/journal.pntd.0006070 (PMC5693297; doi:10.1371/journal.pntd.0006070)
Supplement: S1 Appendix — Description of mathematical framework to estimate airline origin-destination flow between Brazilian airports from publicly available data. (PDF) [file pntd.0006070.s001.pdf]

S1 Appendix. Airline origin-destination flow estimates based on Brazilian airline database

Raquel Martins Lana[1◑], Marcelo Ferreira da Costa Gomes[2*◑], Tiago França Melo de Lima[3], Nildimar Alves Honório[4], Cláudia Torres Codeço[2],

1 Fiocruz, Escola Nacional de Saúde Pública Sérgio Arouca (ENSP), Rio de Janeiro, RJ, Brazil.
2 Fiocruz, Programa de Computação Científica (PROCC), Rio de Janeiro, RJ, Brazil.
3 Laboratório de Engenharia e Desenvolvimento de Sistemas (LEDS), Departamento de Computação e Sistemas (DECSI), Instituto de Ciências Exatas e Aplicadas (ICEA), Universidade Federal de Ouro Preto (UFOP), João Monlevade, MG, Brazil.
4 Fiocruz, Instituto Oswaldo Cruz (IOC), Laboratório de Transmissores de Hematozoários, Rio de Janeiro, RJ, Brazil.

◑These authors contributed equally to this work.
* marcelo.gomes@fiocruz.br

Introduction

The airline data provided by the *Agência Nacional de Aviação Civil* (National Agency for Civil Aviation, ANAC, http://www.anac.gov.br), limits the information about the flow between airport pairs only to the number of passengers on direct flights (with or without stoppage) and the total amount of passengers making a connection in each airport by last boarding airport. Information about these connecting passengers does not include their final destination, that is, in which flight they boarded at the connecting airport. Similarly, data on direct flights do not inform how many passengers took a previous connecting flight before boarding a direct flight. Therefore, for connecting passengers, the data do not provide their final destination, while for passengers on direct flights the data do not inform what the *de facto* airport of origin of each passenger is.

Such limitations create a particular challenge to obtain proper origin-destination matrix between Brazilian airports. Given the continental scale of the Brazilian territory, it is known that in the national grid several routes are made with connecting flights. The availability of direct flights between regions far apart is very limited. The major connection hubs are located in the metropolitan areas of São Paulo, Rio de Janeiro and Brasília, which combine not only densely populated areas but, maybe more importantly, regions that are quite centralized with respect to the North-South axis of the Brazilian territory.

Given the absence of detailed information regarding connecting flights, we propose a method to estimate the number of passengers flying from one state with final destination in another supposing that there is at most one connection in each passenger's route. That is, we assume that the number of passengers that take more than one connection to reach its destination state from its state of origin is significantly lower than those on direct flights or using a single connecting flight. To estimate the number of passengers between two airports with a connecting flight in between, we will combine the information regarding passengers on direct flights and the fraction of passengers on connections in each airport.

To illustrate the proposed method, we will present the general formula and exemplify its usage on a simple network (Fig 1).

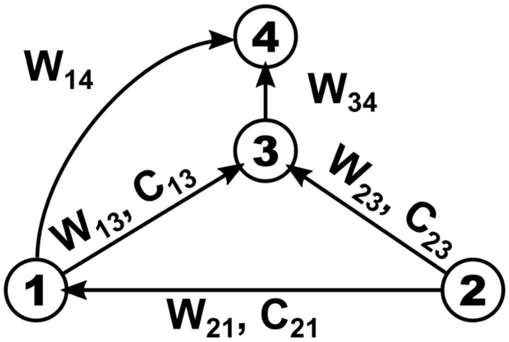

Fig 1. Example of a small directed weighted network with information structure as provided in the Brazilian airline database. Each node represents an airport, and the edges represent passengers flying between them. Weights represent the number of passengers on direct flights from airport i to j (W_{ij}) and passengers from airport i taking a connecting flight at j (C_{ij}).

De facto origin-destination estimate

Let us denote by W_{ij} the number of passengers on a direct flight from i to j, and by C_{ij} those flying from i to make a connection at j. While the former end their trip at j, the later will board another flight at j before finishing his/her trip. Those two quantities are provided for each pair of airports in the Brazilian territory, as described in the previous section. Our goal is to obtain an estimate for the *de facto* origin-destination matrix for the Brazilian flight network given this dataset. That is, we want to estimate what is the actual number of passengers that start their trip at a given airport (origin) and end it at another one (destination), for every pair of airports, which we will denote by Ω_{ij}.

For ease of notation, we will use the dot symbol, "$\cdot$", to indicate sum over indexes when doing so does not compromise readability. For instance, $W_{i\cdot} = \sum_j W_{ij}$ represents the total number of passengers boarding direct flights at i, while $W_{\cdot j} = \sum_i W_{ij}$ denotes the total number of passengers arriving at j on direct flights, that is, with final destination at j.

To estimate the actual flow of passengers from origin i and destination j we will make a few key assumptions:

1. The probability of making a trip with more than one connecting flight is negligible;

2. Passengers on connecting flights are proportionally distributted among the available direct flights at the connecting airport based on the number of passengers in each of these flights.

Proposition 1 implies that the number of passengers on a given origin-destination pair whose trips are made with a direct flight or with at most one connecting flight is much greater than those with more than one connecting flight. Although a strong assumption, it is necessary to limit the number of possible flight combinations between each pair of airports. In the lack of detailed information regarding the typical number of connections, assuming only one is made makes the problem mathematically tractable.

Let us define W^*_{ij} and W^*_{ikj} as the number of passengers whose *origin* is airport i and *destination* is j, through direct flight or with a connection at k, respectively. The difference between W^*_{ij} and W_{ij} is that while the later is the number of passengers on a direct flight from airport i to airport j taken from original data – which has no information on travel origin for each of those passengers, as described –, the former is the estimated number of passengers whose travel origin is airport i and final travel

destination is airport j on a direct flight. That is, W^*_{ij} is our estimate for the *de facto* origin-destination matrix based on direct flights aline. the quantity W^*_{ikj} is the estimated *de facto* origin-destination tensor for passengers with travel origin at i, final destination at j with a connecting flight at k. Finally, Ω_{ij}, the *total number of passengers with travel starting at i and ending at j*, regardless of the path taken, assuming that the number of travelers with more than one connection is negligible, can be approximated by:

$$\Omega_{ij} = W^*_{ij} + \sum_k W^*_{ikj} \ . \tag{1}$$

To estimate the number of passengers from i to j with a connecting flight at k, W^*_{ikj}, let us first define lower case c_{ik} as the ratio between passengers from i making a connection at k with respect to all direct flight passengers at k:

$$c_{ik} = \frac{C_{ik}}{W_{k\cdot}} \ . \tag{2}$$

From this definition, c_{ik} is an estimate of the probability that a passenger boarding at k on a direct flight is, in fact, a connecting passenger originally from i. With proposition 2 we are assuming that passengers arriving at a given airport k for a connecting flight are distributed among the available direct flights from k following a multinomial distribution. Each destination probability is proportional to the number of passengers on each direct flight. Therefore, from the total number of passengers from i making a connection at k – C_{ik} –, and the total number of passengers on a direct flight from k to j – W_{kj} –, the expected number of those that came from i, to make a connection at k, with final destination at j – W^*_{ikj} –, is given by

$$W^*_{ikj} = C_{ik}\frac{W_{kj}}{W_{k\cdot}} = c_{ik}W_{kj} \ . \tag{3}$$

Note that this construction also allows us to estimate the fraction of passengers on direct flight from k to j that are connecting passengers from other airports. In order to estimate the number of passengers who are from k itself – W^*_{kj} – we must take into account all connecting passengers on that flight, $W^*_{\cdot kj}$:

$$W^*_{\cdot kj} = \sum_i c_{ik}W_{kj} = c_{\cdot k}W_{kj} \ . \tag{4}$$

Therefore, the expected number of passengers with origin k and destination j, on a direct flight, is given by

$$W^*_{kj} = W_{kj}\left(1 - c_{\cdot k}\right) \ . \tag{5}$$

Finally, combining Eqs. 3 and 5 with Eq. 1, we have that the expected number of passengers in the origin-destination pair i to j, regardless of route, is

$$\Omega = W_{ij}\left(1 - c_{\cdot i}\right) + \sum_k c_{ik}W_{kj} \ . \tag{6}$$

In order to aggregate this information by state, we sum over the corresponding airports to obtain the estimate for the total number of passengers flying from state I to state J, that is

$$\Omega_{IJ} = \sum_{i \in I} \sum_{j \in J} \Omega_{ij} \; . \tag{7}$$

Since the provided information has a monthly temporal resolution, in order to obtain the average daily flow in month m, defined as $\pi_{IJ,m}$, we divide the estimated flow in that month, $\Omega_{IJ,m}$, by the corresponding number of days in m. In this fashion, we preserve any seasonal effect that might be present at the monthly level, which would otherwise be washed out if flight data was aggregated yearly.

It is important to remind the reader that the assumptions made to obtain these estimates present some limitations. For instance, for any pair of remote airports, the assumption that the number of passengers taking more than one connection is significantly smaller than those taking up to one connection might not hold by sheer lack of available flight paths. Since the flow between remote airports represents a small fraction of interstate airport flow, we believe that the error generated by this simplification does not justify the mathematical complexity of introducing two or more connections in our calculations. Also, the proportional distribution of connecting passengers among direct flights might introduce error. Particularly, this assumption favors the presence of passengers of airport hubs on both origin and destination. This could be addressed by weighting down the number of connecting passengers W^*_{ikj} when the direct flow W_{ij} is high, for instance. Nonetheless, this would only be an issue if C_{ik} is relatively high compared to $C_{\cdot k}$. Since hubs are characterized by having a relatively high presence of direct flights, especially to other hubs, the number of passengers from hub i making a connection on other airports is low compared to the number of passengers on direct flights from that hub.

Example

To exemplify the use of the proposed approximation, we'll make use of the toy network illustrated on Fig. 1. In that network, since there are no passengers on flights to node 2, the number of passengers on direct flights from that node are all originally from that airport. From Eq. 5 this means that $W^*_{21} = W_{21}$ and $W^*_{23} = W_{23}$. Since connecting passengers from node 2 have connections at nodes 1 and 3, and there is no direct flight from 3 to 1, the only possible route from node 2 with final destination at 1 is via direct flight. Therefore, the flow from node 2 to nodes 1 is simply

$$2 \to 1 : \Omega_{21} = W_{21} \tag{8}$$

Regarding the flow of passengers with final destination at node 3, we have the following estimates for passengers with origin at nodes 1 and 2 becomes more involved. On the one hand, since there are no flights bound to node 2, all passengers boarding at that node are necessarily from there, giving $W^*_{23} = W_{23}$. On the other hand, for passengers from 1 to 3 we have not only the local population of 1 but also connecting passengers arriving at 1 from flights originated at node 2, given by C_{21}. Therefore, for passengers arriving on a direct flight from node 1 to node 3, W_{13}, we have passengers originally from 1 but also from 2. This leads to the following estimate for the origin-destination flow to node 3:

$$1 \to 3 : \Omega_{13} = W^*_{13} = W_{13}\left(1 - \frac{C_{21}}{W_{13} + W_{14}}\right) \ , \qquad (9)$$

$$2 \to 3 : \Omega_{23} = W^*_{23} + W^*_{213} = W_{23} + W_{13}\frac{C_{21}}{W_{13} + W_{14}} \ . \qquad (10)$$

Taking node 4 as final destination, from node 1 we would have both passengers on direct flight and with connection at 3. From node 2, the only possibility is via connecting flights, either at 1 or 3. From node 3, there are only passengers to 4 trough direct flight. While the direct flight from node 1 to 4 can carry passengers from nodes 1 and 2, direct flight from node 3 to 4 can carry passengers originally from nodes 1, 2 and 3. Combining all this information, we end up with the following set of equations for the estimate for each node of origin:

$$\begin{aligned} 1 \to 4 : \Omega_{14} &= W_{14}\left(1 - \frac{C_{21}}{W_{13} + W_{14}}\right) + W_{34}\frac{C_{13}}{W_{34}} \ , \\ : \Omega_{14} &= W_{14}\left(1 - \frac{C_{21}}{W_{13} + W_{14}}\right) + C_{13} \ , \end{aligned} \qquad (11)$$

$$2 \to 3 : \Omega_{23} = \frac{C_{21}}{W_{13} + W_{14}} + C_{23} \ , \qquad (12)$$

$$3 \to 4 : \Omega_{34} = W_{34}\left(1 - \frac{C_{13} + C_{23}}{W_{34}}\right) \ . \qquad (13)$$
