# Supplementary material for: The introduction of dengue follows transportation infrastructure changes in the state of Acre, Brazil: A network-based analysis
Source: PLoS Negl Trop Dis. 2017 Nov 17;11(11):e0006070. doi: 10.1371/journal.pntd.0006070 (PMC5693297; doi:10.1371/journal.pntd.0006070)
Supplement: S2 Appendix — Full matrix of centrality measures for each Acrean Municipality and confusion matrix of those measures. (PDF) [file pntd.0006070.s002.pdf]

PLOS | NEGLECTED TROPICAL DISEASES

# Supplementary file 2: Network descriptors and the correlations between them.

## S2 Table 1 - Network descriptors values for Acrean municipalities.

| Acronym | Municipality | Sw | Ew | Dw | Dk | Bw | Cw | B | D | C |
|---|---|---|---|---|---|---|---|---|---|---|
| RB | Rio Branco | 0.254 | 0.672 | 0.000 | 0.000 | 0.505 | 103.849 | 0.631 | 0.000 | 0.396 |
| SG | Senador Guiomard | 0.105 | 0.521 | 0.443 | 0.035 | 0.000 | 98.183 | 0.414 | 1.000 | 0.344 |
| Brl | Brasiléia | 0.084 | 0.094 | 0.212 | 0.004 | 0.024 | 67.162 | 0.095 | 0.250 | 0.196 |
| SM | Sena Madureira | 0.034 | 0.137 | 0.272 | 0.007 | 0.024 | 83.239 | 0.152 | 0.500 | 0.280 |
| Epcd | Epitaciolândia | 0.065 | 0.079 | 0.193 | 0.004 | 0.000 | 63.542 | 0.181 | 0.333 | 0.236 |
| Xpr | Xapuri | 0.025 | 0.067 | 0.209 | 0.005 | 0.000 | 63.074 | 0.000 | 0.333 | 0.226 |
| Cpb | Capixaba | 0.024 | 0.101 | 0.229 | 0.012 | 0.024 | 67.958 | 0.338 | 0.500 | 0.288 |
| Acld | Acrelândia | 0.018 | 0.071 | 0.229 | 0.008 | 0.005 | 67.827 | 0.033 | 1.000 | 0.296 |
| PC | Plácido de Castro | 0.035 | 0.151 | 0.237 | 0.009 | 0.014 | 76.340 | 0.014 | 0.500 | 0.266 |
| CZS | Cruzeiro do Sul | 0.103 | 0.194 | 0.284 | 0.001 | 0.167 | 91.441 | 0.417 | 1.000 | 0.356 |
| AB | Assis Brasil | 0.019 | 0.025 | 0.161 | 0.003 | 0.005 | 53.824 | 0.000 | 0.200 | 0.165 |
| PA | Porto Acre | 0.053 | 0.298 | 0.354 | 0.016 | 0.000 | 93.311 | 0.000 | 1.000 | 0.288 |
| Bjr | Bujari | 0.038 | 0.223 | 0.327 | 0.039 | 0.000 | 90.875 | 0.202 | 1.000 | 0.323 |
| Fj | Feijó | 0.020 | 0.070 | 0.225 | 0.003 | 0.000 | 67.016 | 0.069 | 0.250 | 0.233 |
| Trc | Tarauacá | 0.017 | 0.054 | 0.214 | 0.002 | 0.076 | 63.651 | 0.179 | 0.333 | 0.269 |
| MU | Manoel Urbano | 0.006 | 0.020 | 0.177 | 0.004 | 0.000 | 36.062 | 0.145 | 0.333 | 0.250 |
| RA | Rodrigues Alves | 0.042 | 0.080 | 0.180 | 0.002 | 0.000 | 82.303 | 0.205 | 0.500 | 0.309 |
| ML | Mâncio Lima | 0.037 | 0.085 | 0.199 | 0.001 | 0.000 | 79.606 | 0.000 | 0.500 | 0.288 |
| SRP | Santa Rosa do Purus | 0.003 | 0.010 | 0.157 | 0.002 | 0.000 | 21.106 | 0.000 | 0.250 | 0.202 |
| MT | Marechal Thaumaturgo | 0.005 | 0.008 | 0.136 | 0.001 | 0.005 | 31.656 | 0.000 | 0.333 | 0.216 |
| PW | Porto Walter | 0.009 | 0.018 | 0.144 | 0.001 | 0.000 | 58.011 | 0.095 | 0.500 | 0.273 |
| Jrd | Jordão | 0.002 | 0.001 | 0.128 | 0.001 | 0.000 | 25.571 | 0.000 | 0.250 | 0.214 |

**B**: betweenness

**C**: closeness

**Bw**: weighted betweenness

**Cw**: weighted closeness

**Ew**: eigenvector

**Sw**: strength

**D**: network distance from Rio Branco in degrees

**Dw**: effective distance from Rio Branco

**Dk**: network distance from Rio Branco in kilometers

<u>Heatmap</u>

To evaluate how correlated the network descriptors are to each other, a heatmap was constructed ranging from beige tones to brown (maximum correlation = 1). We can see a strong correlation between topological distance from Rio Branco, measured by effective and degree distance, and node relevance to information flow in the network, measured by eigenvector and closeness centrality. Geographical distance, although also showing positive correlation is not so strong. This highlights that human mobility is not only dependant on distance, but also on ease of access and infrastructure. Likewise, weighted betweenness centrality also does not have a strong signal with other measures. This might be due to the relatively small size of the network.

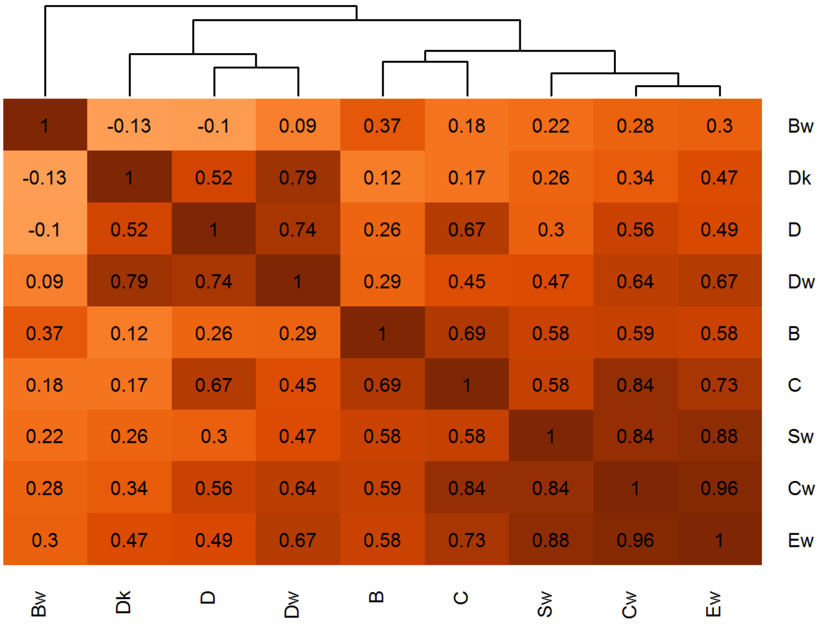


**S2 Fig 1. Heatmap of the spearman correlation between node centrality measures used on the structural and mobility networks.**
