# Supplementary material for: The introduction of dengue follows transportation infrastructure changes in the state of Acre, Brazil: A network-based analysis
Source: PLoS Negl Trop Dis. 2017 Nov 17;11(11):e0006070. doi: 10.1371/journal.pntd.0006070 (PMC5693297; doi:10.1371/journal.pntd.0006070)
Supplement: S1 Fig — Vectorial format of the structural network of Acrean municipalities for each dengue epidemiological year from 2000/2001 to 2014/2015. (PDF) [file pntd.0006070.s003.pdf]

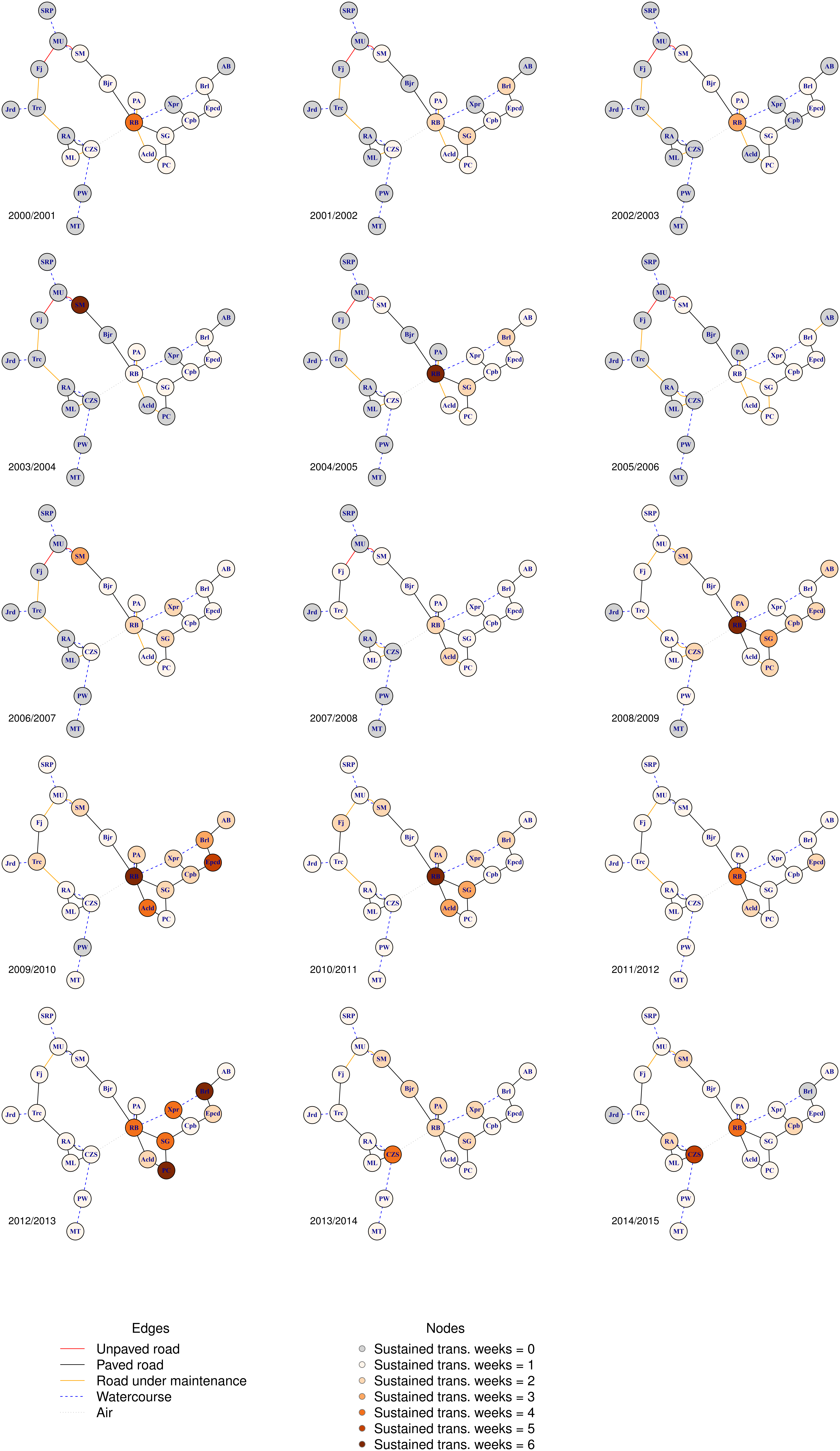
2000/2001
2001/2002
2002/2003
2003/2004
2004/2005
2005/2006
2006/2007
2007/2008
2008/2009
2009/2010
2010/2011
2011/2012
2012/2013
2013/2014
2014/2015
SRP
MU
SM
Fj
Bjr
AB
Brl
PA
Xpr
Epcd
Jrd
Trc
Cpb
RB
SG
RA
CZS
ML
Acld
PC
PW
MT
Edges
Unpaved road
Paved road
Road under maintenance
Watercourse
Air
Nodes
Sustained trans. weeks = 0
Sustained trans. weeks = 1
Sustained trans. weeks = 2
Sustained trans. weeks = 3
Sustained trans. weeks = 4
Sustained trans. weeks = 5
Sustained trans. weeks = 6
